# Associations of genetic variation and mRNA expression of PDGF/PDGFRB pathway genes with coronary artery disease in the Chinese population

**DOI:** 10.1111/jcmm.70193

**Published:** 2024-11-21

**Authors:** Pengfei Wei, Hankun Xie, Junxiang Sun, Qian Zhuang, Jichao Xie, Yunjie Yin, Fangyuan Liu, Wen Li, Changying Chen, Feifan Wang, Xu Han, Liang Xu, Xianghai Zhao, Yanchun Chen, Song Yang, Chong Shen

**Affiliations:** ^1^ Department of Cardiology Affiliated Yixing People's Hospital of Jiangsu University, People's Hospital of Yixing City Yixing China; ^2^ Department of Epidemiology, Center for Global Health, School of Public Health Nanjing Medical University Nanjing China

**Keywords:** case–control study, coronary artery disease, genetic variation, mRNA expression, PDGF pathway

## Abstract

Platelet‐derived growth factors (PDGFs) and receptors (PDGFR) play a key role in the process of coronary atherosclerosis. We aimed to investigate the association of genetic variations and mRNA expressions of PDGF/PDGFRB pathway genes with coronary artery disease (CAD). In this case–control study (3139 CAD vs. 3270 controls), 13 single nucleotide polymorphisms (SNPs) at five pathway genes were genotyped and combined to construct a weighted genetic risk score (wGRS). Three hundred and six pairs of cases and controls were selected for mRNA quantification. Restricted cubic spline (RCS) analyses were conducted for the dose–response relationship between wGRS, mRNAs and CAD. Area under the curve (AUC) was estimated to evaluate the discrimination of wGRS, mRNAs, and traditional risk factors (TRF) for CAD. The wGRS exhibited a positive linear relationship with CAD (*p* for linearity <0.001), and the medium and high wGRS had 37% and 50% increased risk of CAD compared to the low wGRS group (*p* = 1.5 × 10^−4^; *p* = 5.7 × 10^−5^). mRNA expression levels of five genes in peripheral blood leukocytes were all lower among patients at admission than controls (*p* < 0.001). The PDGF/PDGFRB mRNA expressions had significant non‐linear correlations with AMI, with “U”‐shaped trend for *PDGFA*, *PDGFB* and “L”‐shaped trend for *PDGFC*, *PDGFD* and *PDGFRB*. Adding wGRS and mRNAs to the TRF model significantly improved the discrimination for CAD with an AUC of 0.921 (95% CI, 0.898–0.943). Genetic variations in the PDGF/PDGFRB pathway contribute to CAD susceptibility with a significantly joint effect. The down‐regulated PDGF/PDGFRB mRNAs in peripheral leukocytes have the potential as blood‐based biomarkers for CAD with high discriminative value.

## 1 INTRODUCTION

Cardiovascular disease remains the leading cause of premature death and disease burden in China and worldwide.^1^ Coronary artery disease (CAD) is a major contributor to the rising morbidity and mortality of cardiovascular disease.^2^, ^3^ In recent decades, there is an growing trend for the incidence and death rate of CAD in China, affecting approximately 11.39 million sufferers.^4^ Acute myocardial infarction (AMI) is the most severe and critical manifestation of CAD with a high disability rate and poor prognosis. Thus, it is of great significance to conduct research on the biomarker identification, mechanism comprehension and population prevention for CAD.

CAD occurs due to the stenosis or occlusion of coronary arteries caused by atherosclerotic plaques or thrombosis, which are affected by the interaction of genetic and environmental factors.^5^ Numerous cytokines participate in the atherosclerotic pathogenesis through different pathophysiological pathways, which may be involved in CAD development and progression.^6^, ^7^ The platelet‐derived growth factor (PDGF) family has been extensively studied since the 1970s, consisting of four members: PDGF‐A, PDGF‐B, PDGF‐C and PDGF‐D. PDGFs exert their biological functions through two PDGF receptor (PDGFR) tyrosine kinases, PDGFR‐α and PDGFR‐β, thus forming the PDGF/PDGFR signalling pathway.^8^ PDGFs and their receptors are critical regulators of the cardiovascular system with functions of cardiac angiogenesis, wound healing, monocyte differentiation, macrophage migration, as well as stimulating mitogenicity and chemotaxis of fibroblasts and smooth muscle cells.^9^, ^10^ So, the PDGF/PDGFR pathway plays a key role in the process of coronary atherosclerosis, which has been supported by in vivo and in vitro studies.^11^, ^12^

Genetic variations that occur naturally contribute to a certain risk of CAD. Genome‐wide association studies (GWAS) to date have identified over 300 major susceptibility loci for CAD.^13^ Among the GWAS‐identified loci, the locus encoding *PDGFD* was detected to be associated with CAD risk, which has been supported by fine mapping.^14^ Nonetheless, common variants achieving genome‐wide significance explain only a small fraction of the CAD heritability and the effect of a single SNP on disease is very weak. However, evidence suggests that weakly associated variants may provide important biological information about the disease when such variants cluster within functional pathways, and the joint contributions of variants with modest effects need to be paid more attention.^15^

Previous studies, through Microarray or RNA sequencing, have identified differentially expressed genes and biological pathways significantly associated with CAD.^16^, ^17^, ^18^ Research also indicate that peripheral blood RNA expressions of functional genes could serve as potential biomarkers for CAD prediction and diagnosis.^19^, ^20^, ^21^ A study found that the expression of PDGF pathway genes in peripheral monocytes correlated with coronary collateral circulation in patients with CAD.^22^ In addition, results of an animal study showed that mRNA expressions of PDGFA, PDGFB, PDGFC, PDGFD and PDGFRs exhibited different changes in the infarcted rat heart in the early and late stages after myocardial infarction.^23^ These findings provide transcriptomic evidence for the role of PDGF/PDGFR pathway in the early occurrence and progression of CAD, which warrants exploration and validation in population research.

We conducted this study to investigate the association of genetic variations and mRNA expressions of PDGF/PDGFRB pathway genes with CAD in a Chinese Han population. Then we evaluated the discriminative value of weighted genetic risk score (wGRS) and *PDGF/PDGFRB* mRNAs in peripheral blood leukocytes for CAD.

## 2 METHODS

### 2.1 Study design and participants

This case–control study consisted of 3139 hospital‐sourced CAD patients and 3270 matched controls from community‐based cohort population. All cases were newly diagnosed with CAD, admitted to the Yixing People's Hospital (Jiangsu, China) from February 2009 to December 2021 and aged 35–80 years at admission. CAD was determined based on coronary angiography with stenosis degree ≥50% in any section of coronary arteries according to the “Guideline on the diagnosis and treatment of stable coronary artery disease” released by the Chinese Society of Cardiology.^24^ Exclusion criteria included repeated hospitalization, aortic dissection, severe aortic valve stenosis, aorto‐arteritis, atrial fibrillation accompanied with coronary embolism, severe hepatic or renal insufficiency, and malignancy. Each control was randomly selected from a subset of candidate cohort subjects following the matching criteria that they had the same gender as the case and age difference within the 2‐year range, and without the diagnosis history of CAD at any time, any cardiac disease or stroke, severe hepatic or renal insufficiency, or malignancy. The cohort studies were conducted in Yixing City, Jurong City and Suqian City (Jiangsu, China), and baseline data were collected from 2009 to 2019. All the cohort subjects were recruited either by cluster random sampling, or by a multi‐stage sampling approach combining probability‐proportional‐to‐size sampling and random sampling from the communities.

From the total population, we selected 306 pairs of AMI cases and controls for mRNA experiments, and 250 samples of the cases at discharge after admitted 5–7 days were also included in the study experiments and analyses. The diagnosis of AMI followed the Fourth Universal Definition of Myocardial Infarction (2018).^25^ Subjects taking anti‐platelet agents and statins in the past 2 weeks, with bleeding disorders or active bleeding, participating in other interventional clinical trials were not within our selection.

This study adhered to principles of the Helsinki Declaration and received approval from the Ethics Committee of Nanjing Medical University in Jiangsu Province of China (2015077; 2018675). All subjects provided written informed consent before participating in the study.

### 2.2 Population survey and clinical measurements

We collected data on demographic characteristics, personal behaviours and habits, disease status and history, medications, blood pressure and biochemical indices for all CAD cases and controls. Information of CAD patients were extracted from medical records in the hospital and de‐identified, which were recorded by physicians at the time of admission. Data of controls were obtained from the baseline survey of cohort studies, including questionnaire interviews, physical examinations, anthropometric measurements and laboratory tests. Smoking was defined as having at least 20 cigarettes per week for a minimum duration of 3 months within a year. Drinking was defined as alcohol consumption at least twice per week for a minimum duration of 6 months a year.^26^ Systolic blood pressure (SBP) and diastolic blood pressure (DBP) were measured at least three times with calibrated instruments. Hypertension was defined as self‐reported diagnosis of hypertension, or currently taking anti‐hypertensive medications, or SBP ≥140 mmHg or DBP ≥90 mmHg. Diabetes was defined as self‐reported diagnosis of diabetes, or current usage of hypoglycemic agents, or fasting glucose ≥7.0 mmol/L.^27^ Dyslipidemia was defined as abnormal changes of lipid indices (total cholesterol [TC] ≥5.2 mmol/L, triglyceride [TG] ≥1.7 mmol/L, low‐density lipoprotein cholesterol [LDL‐C] ≥3.4 mmol/L, or high‐density lipoprotein cholesterol [HDL‐C] <1.04 mmol/L), or having diagnosed dyslipidemia history, or currently taking lipid‐lowering drugs.^28^

Peripheral venous blood samples were collected into the vacuum anticoagulation tube with ethylene diamine tetraacetic acid dipotassium (EDTA‐K2) for cases at admission and for controls at baseline after an overnight fasting. All samples were processed within 2 h, and then transported via the cold chain within 24 h for laboratory tests or stored at −20°C for subsequent experiments. Laboratory tests included the measurements of serum glucose, TC, TG, HDL‐C and LDL‐C levels.

### 2.3 Selection of tagging single nucleotide polymorphisms and genotyping

With the pathway genes *PDGFA*, *PDGFB*, *PDGFC*, *PDGFD* and *PDGFRB* as candidate genes, we searched variants from the upstream 2 kb to the downstream 1 kb and selected tagging single nucleotide polymorphisms (tagSNPs) based on data of the Han Chinese in Beijing, China (CHB) population in the 1000 Genomes Project (GRCh37, http://phase3browser.1000genomes.org/index.html) and the International HapMap Project. SNPs with the minor allele frequency (MAF) ≥5%, linkage disequilibrium (LD) of *R* ^2^ ≤ 0.8, and predicted to have biological functions (http://snpinfo.niehs.nih.gov/snpinfo/snpfunc.htm, http://www.regulomedb.org/) were taken into consideration. Finally, 13 tagSNPs in five PDGF/PDGFRB pathway genes were selected in this study. Genetic information and functional prediction of these 13 tagSNPs are listed in Table S1.

DNA was isolated from peripheral blood using the protein precipitation method (Eaglink, EGEN2024, China), and then measured with the NanoDrop 2000 spectrophotometer (Thermo Fisher Scientific, Waltham, MA). Genotyping of SNPs was performed by the polymerase chain reaction (PCR) TaqMan MGB probe assay on the platform of ABI 9700 PCR system (Applied Biosystems, Foster City, CA). Results were read on the 7900HT real‐time PCR system (Applied Biosystems, Foster City, CA) with the Sequence Detection System (SDS) software 2.4. The sequences of primers and probes for SNP genotyping are shown in Table S2. Thirteen SNPs were successfully genotyped, of which the allele and genotype frequency were all consistent with the Hardy–Weinberg equilibrium (*p* > 0.05) in the population of controls (Table S1).

### 2.4 RNA extraction and mRNA quantification

Leukocytes were separated from peripheral whole blood samples through gradient centrifugation (3800 rpm, 5 min), mixed with RNA preservative (Eaglink, EGEN2026, China), and stored at −20°C. Total RNA was extracted from peripheral leukocytes using the RNA extraction kit based on the magnetic bead method (Yuan, Yu‐BR02‐1, China), which was then measured by the NanoDrop 2000 spectrophotometer (Thermo Fisher Scientific, Waltham, MA). Reverse transcription of RNA was carried out using the PrimeScript RT Reagent Kit with gDNA Eraser (TaKaRa, RR047A, Japan). Newly‐synthesized cDNA was diluted in nuclease‐free water and stored at −80°C or used immediately.

mRNA expressions of *PDGFA*, *PDGFB*, *PDGFC*, *PDGFD* and *PDGFRB* in leukocytes were relatively quantified via the SYBR Green real‐time quantitative PCR (RT‐qPCR) assay with the housekeeping gene Glyceraldehyde‐3‐Phosphate Dehydrogenase (*GAPDH*) as internal reference. Specific primers of six genes were designed online (https://www.ncbi.nlm.nih.gov/tools/primer‐blast/) and synthetized by Sangon Biotech Co., Ltd. (Shanghai, China) (Table S3). The RT‐qPCR reaction system included cDNA samples, forward and reverse primers, Platinum SYBR Green qPCR SuperMix‐UDG (Invitrogen, 11,733‐046, USA), and RNase‐free dH_2_O. RT‐qPCR reactions were performed on the FS384 Real‐Time PCR System platform (Fusheng Biotechnology, China), following procedures of incubation at 95°C for 5 min, and 40 cycles of denaturation at 95°C for 10 s, annealing at 57°C for 20 s, extension at 72°C for 20 s. All samples were tested in triplicate in the 384‐well plate, and cycle threshold (CT) values were recorded. The 2^−ΔΔCT^ method was used to calculate mRNA expression levels of target genes relative to the reference gene.^29^

### 2.5 Statistical analysis

Categorical variables were presented as numbers (percentages) and analysed by the Chi‐squared (*χ* ^2^) test. Continuous variables were presented as medians and interquartile range (IQR) for the non‐normality of distribution. The Mann–Whitney *U* test was performed to examine differences in population characteristics and mRNA expression levels between cases and controls. The Wilcoxon signed rank test was used for paired comparison between cases at admission and at discharge. Fold change was calculated as the ratio of mRNA levels in two groups. The Hardy–Weinberg equilibrium for SNPs in the study population were tested with Fisher exact *χ* ^2^. The Kruskal–Wallis test was used for the comparison of mRNA expression levels among SNP genotypes, with the Jonckheere–Terpstra test for trend analyses.

Multivariable logistic regression was applied to assess the association between SNPs and CAD in the whole population and in subgroup analyses. The wGRS was constructed for CAD by summing the multiplication value of risk allele numbers (0/1/2) and corresponding weights of 13 SNPs. The weight of each SNP was *β* estimate generated from the logistic regression analysis of single SNP and CAD in additive models (wild type vs. heterozygote type vs. mutant type) with covariates adjusted. The wGRS was then categorized into three groups: low risk (lowest quintile), medium risk (2nd–4th quintiles), and high risk (highest quintile). The association of wGRS and wGRS groups with CAD was evaluated by adjusted and unadjusted logistic models. Restricted cubic spline (RCS) regression with four knots^30^, ^31^ was used to plot the dose–response relationship of wGRS and *PDGF/PDGFRB* mRNA expressions with the risk of CAD. Based on RCS results, we divided the expression levels of five genes into quartiles, namely low, medium‐low, medium‐high, and high groups, with the high group (4th quartile) as reference in following logistic analyses of mRNAs and AMI. Multivariable regression models were adjusted for age, gender, smoking, drinking, hypertension, diabetes and dyslipidemia. The odds ratio (OR) and 95% confidence intervals (CIs) of logistic regression analyses were reported.

Spearman coefficients were calculated for the correlation between wGRS and mRNA expression levels in controls, cases at admission and at discharge. Area under the curve (AUC) of the receiver operating characteristic (ROC) and 95% CI were estimated to evaluate the discrimination performance of wGRS, mRNAs and traditional risk factors (TRF) for CAD, and DeLong test was used for AUC comparisons among different models. TRF included age, gender, smoking, drinking, hypertension, diabetes and dyslipidemia.

Statistical analyses were carried out using R version 4.2.1 (R Foundation for Statistical Computing, Vienna, Austria) and SAS software 9.4 (SAS Institute Inc., Cary, NC, USA). Analysis results were considered statistically significant at the two‐sided 5% level. False discovery rate (FDR) adjustments were applied for multiplicity of testing.

## 3 RESULTS

### 3.1 Characteristic description of cases and controls

Table 1 presents the participant characteristics of this case–control study. A total of 3139 CAD patients and 3270 controls were included for genetic analyses. Age of controls were comparable to that of cases, with the median of 64 years in both groups (*p* = 0.819). The gender proportion were also similar in two groups, with 74.4% males in cases and 73.4% in controls (*p* = 0.397). There was no difference in smoking status and glucose levels between cases and controls, while significant differences existed regarding the prevalence of drinking, hypertension, diabetes, dyslipidemia and the level of blood pressure and lipids (all *p* < 0.001).

**TABLE 1:** Population characteristics of the case–control study.

Characteristics	Total population for genetic analysis				Subset for mRNA analysis			
	CAD cases (*n* = 3139)	Controls (*n* = 3270)	*Z*/*χ* ^2^	*p‐*value	AMI cases (*n* = 306)	Controls (*n* = 306)	*Z/χ* ^2^	*p‐*value
Age (years), median (IQR)	64 (55, 71)	64 (56, 70)	0.229	0.819	66 (57, 73)	65 (56, 71)	1.441	0.150
Age group, *n* (%)			0.431	0.512			0.419	0.518
<65 years	1649 (52.5)	1690 (51.7)			144 (47.1)	152 (49.7)		
≥65 years	1490 (47.5)	1580 (48.3)			162 (52.9)	154 (49.3)		
Gender, *n* (%)			0.716	0.397			0.155	0.693
Female	805 (25.6)	870 (26.6)			63 (20.6)	68 (22.2)		
Male	2334 (74.4)	2400 (73.4)			243 (79.4)	238 (77.8)		
Smoking, *n* (%)			0.452	0.502			14.834	<0.001
No	1819 (57.9)	1923 (58.8)			154 (50.3)	202 (66)		
Yes	1320 (42.1)	1347 (41.2)			152 (49.7)	104 (34)		
Drinking, *n* (%)			438.782	<0.001			49.235	<0.001
No	2644 (84.2)	1987 (60.8)			268 (87.6)	192 (62.7)		
Yes	495 (15.8)	1283 (39.2)			38 (12.4)	114 (37.3)		
Hypertension, *n* (%)			262.882	<0.001			13.256	<0.001
No	1050 (33.5)	1752 (53.6)			124 (40.5)	170 (55.6)		
Yes	2089 (66.5)	1518 (46.4)			182 (59.5)	136 (44.4)		
Diabetes, *n* (%)			397.487	<0.001			29.883	<0.001
No	2156 (68.7)	2910 (89.0)			214 (69.9)	270 (88.2)		
Yes	983 (31.3)	360 (11.0)			92 (30.1)	36 (11.8)		
Dyslipidemia, *n* (%)			1865.743	<0.001			0.085	0.771
No	1515 (48.3)	3150 (96.3)			301 (98.4)	299 (97.7)		
Yes	1624 (51.7)	120 (3.7)			5 (1.6)	7 (2.3)		
SBP (mmHg), median (IQR)	130 (120, 146)	140 (128, 154)	17.384	<0.001	122 (108, 135)	145.17 (130.75, 160)	13.017	<0.001
DBP (mmHg), median (IQR)	80 (71, 87)	82 (75, 89)	10.819	<0.001	72 (66, 80)	84 (76.08, 92)	11.069	<0.001
GLU (mmol/L), median (IQR)	5.43 (4.71, 7.03)	5.49 (5.05, 6.15)	1.815	0.070	6.10 (5.31, 7.92)	5.76 (5.38, 6.44)	2.770	0.006
TC (mmol/L), median (IQR)	4.29 (3.58, 5.02)	4.76 (4.17, 5.37)	18.522	<0.001	4.52 (3.90, 5.21)	4.72 (4.14, 5.36)	2.629	0.009
TG (mmol/L), median (IQR)	1.43 (1.01, 2.10)	1.33 (0.93, 1.97)	4.858	<0.001	1.41 (1.04, 2.05)	1.38 (0.96, 1.89)	0.990	0.322
HDL‐C (mmol/L), median (IQR)	1.05 (0.92, 1.22)	1.31 (1.09, 1.56)	31.174	<0.001	1.07 (0.95, 1.23)	1.29 (1.07, 1.57)	8.630	<0.001
LDL‐C (mmol/L), median (IQR)	2.51 (1.97, 3.07)	2.74 (2.26, 3.26)	11.420	<0.001	2.85 (2.34, 3.36)	2.80 (2.34, 3.38)	0.452	0.651

*Note*: Data are presented as numbers (column percentages) or the median (interquartile range). *Z* was from the Mann–Whitney *U* test. *χ* ^2^ was from the Chi‐squared test.

Abbreviations: AMI, acute myocardial infarction; CAD, coronary artery disease; DBP, diastolic blood pressure; GLU, glucose; HDL‐C, high‐density lipoprotein cholesterol; IQR, interquartile range; LDL‐C, low‐density lipoprotein cholesterol; SBP, systolic blood pressure; TC, total cholesterol; TG, triglycerides.

In the mRNA‐related analysis, age and gender were both matched in this subset of study population. The median age of cases and controls were 66 and 65 years (*p* = 0.150), and the male percentages were 79.4% and 77.8%, respectively (*p* = 0.693). No difference existed in the dyslipidemia prevalence, TG and LDL‐C levels between cases and controls, but differences were detected for the proportion of smoking, drinking, hypertension, diabetes, and the level of blood pressure, glucose, TC and HDL‐C (all *p* < 0.01).

### 3.2 Association analyses of PDGF/PDGFRB pathway SNPs and CAD

Among 13 tagSNPs in the PDGF/PDGFRB pathway, only *PDGFRB* variant rs246390 (A > G) was significantly associated with a decreased risk of CAD. Adjusted OR for the additive model was 0.89 (95% CI, 0.81–0.97; *p* = 0.009) (Table S4). This association did not reach statistical significance after FDR adjustment (*P*_*FDR*_ = 0.063).

We also did subgroup analyses for the association between pathway SNPs and CAD by age, gender, smoking, drinking, hypertension, diabetes and dyslipidemia (Tables S5–S7). When stratified by these factors, the association between genetic variation of rs246390 and CAD was still significant in the younger (<65 years) (OR, 0.87 [95% CI, 0.76–0.99]), female (OR, 0.82 [95% CI, 0.69–0.98]), and drinker (OR, 0.81 [95% CI, 0.68–0.97]) subgroups, and in the populations with hypertension, diabetes, without dyslipidemia (*p* = 0.016; *p* = 0.005; *p* = 0.014). Associations also existed between CAD and SNP rs5757573 C >T at *PDGFB* in males and smokers (*p* = 0.030; *p* = 0.021), SNP rs342309 G >A at *PDGFC* in males (*p* = 0.026), SNP rs13053714 G >A at *PDGFB* in females (*p* = 0.028), and SNP rs3828610 A >C at *PDGFRB* among drinkers (*p* = 0.009). Besides, *PDGFRB* variants rs3828610 and rs9324641 had significant relationship with a higher risk of CAD in the subgroup with dyslipidemia (*p* = 0.009; *p* = 0.029).

### 3.3 Association between wGRS and the risk of CAD

**FIGURE 1:**
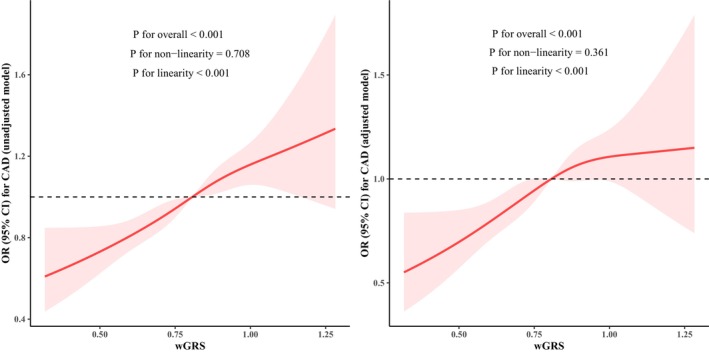
Restricted cubic spline regression of the weighted genetic risk score and coronary artery disease. ORs are indicated by solid lines and 95% CIs by shaded areas, which were estimated by logistic regression analyses. Adjusted model: Adjusted for age, gender, smoking, drinking, hypertension, diabetes, and dyslipidemia. CAD, coronary artery disease; CI, confidence interval; OR, odds ratio; wGRS, weighted genetic risk score.

Risk alleles and specific weights of 13 pathway SNPs for CAD from multivariable logistic regression analyses are listed in Table S8, which were used to calculate wGRS. The distribution and Kernel density for wGRS was plotted in Figure S1, with the mean 0.80 and standard deviation 0.17. Table 2 shows the association between wGRS and the risk of CAD in different models. Compared to individuals in the lowest quintile of wGRS, those with medium and high wGRS had significantly higher risk of CAD in both unadjusted and adjusted models, with unadjusted ORs of 1.30 (95% CI, 1.15–1.48; *p* = 4.9 × 10^−5^) and 1.54 (95% CI, 1.32–1.80; *p* = 5.8 × 10^−8^), adjusted ORs of 1.37 (95% CI, 1.16–1.61; *p* = 1.5 × 10^−4^) and 1.50 (95% CI, 1.23–1.83; *p* = 5.7 × 10^−5^). RCS analyses of wGRS and CAD in Figure 1 displayed that wGRS had a positive linear relationship with the risk of CAD in unadjusted and adjusted models (both *p* for linearity <0.001).

**TABLE 2:** Association of weighted genetic risk score (wGRS) with the risk of coronary artery disease.

	Model 1		Model 2		Model 3	
	OR (95% CI)	*p‐*value	OR (95% CI)	*p‐*value	OR (95% CI)	*p‐*value
wGRS, continuous	2.416 (1.812–3.225)	2.0 × 10^−9^	2.416 (1.812–3.225)	2.0 × 10^−9^	2.284 (1.59–3.285)	8.0 × 10^−6^
wGRS, low	Reference	–	Reference	–	Reference	–
wGRS, medium	1.302 (1.147–1.479)	4.9 × 10^−5^	1.302 (1.147–1.479)	4.9 × 10^−5^	1.366 (1.164–1.606)	1.5 × 10^−4^
wGRS, high	1.540 (1.318–1.800)	5.8 × 10^−8^	1.539 (1.317–1.799)	6.1 × 10^−8^	1.500 (1.231–1.828)	5.7 × 10^−5^

*Note*: Model 1: unadjusted. Model 2: adjusted for age and gender. Model 3: adjusted for age, gender, smoking, drinking, hypertension, diabetes, dyslipidemia. wGRS categories: low risk (lowest quintile of wGRS), medium risk (2nd–4th quintiles of wGRS), and high risk (highest quintile of wGRS).

Abbreviations: CI, confidence interval; OR, odds ratio.

### 3.4 *PDGF* / *PDGFRB* mRNA expression comparison between AMI cases and controls

**FIGURE 2:**
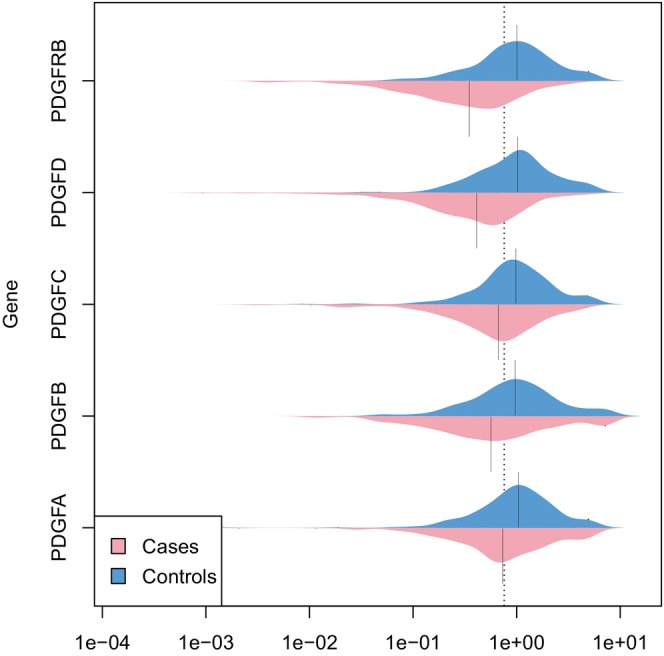
Distribution of the PDGF/PDGFRB mRNA expression in cases and controls. Vertical black lines in the middle of plots are medians. Mann–Whitney *U* test was used for mRNA comparisons between cases and controls. The mRNA expression levels of five genes in cases were all significantly lower than those in controls (all *p* < 0.001). PDGFA, platelet‐derived growth factor A; PDGFB, platelet‐derived growth factor B; PDGFC, platelet‐derived growth factor C; PDGFD, platelet‐derived growth factor D; PDGFRB, platelet‐derived growth factor receptor β.

The mRNA expression levels of genes *PDGFA*, *PDGFB*, *PDGFC*, *PDGFD* and *PDGFRB* in AMI cases at admission were all significantly lower than those in controls, with the fold change of 0.71, 0.59, 0.68, 0.40 and 0.35 (all *p* < 0.001) (Table S9). The distribution of the five gene mRNA expression in cases and controls were compared in Figure 2. Comparing the *PDGF/PDGFRB* mRNA expression levels between AMI cases at admission and at discharge, we found that the expression of *PDGFB*, *PDGFC*, *PDGFD* and *PDGFRB* were significantly up‐regulated as the patients discharged (*p* = 0.002 for *PDGFB*; *p* < 0.001 for *PDGFC*, *PDGFD* and *PDGFRB*) (Table S10).

### 3.5 Association of *PDGFs* and *PDGFRB* mRNA expression with AMI

**FIGURE 3:**
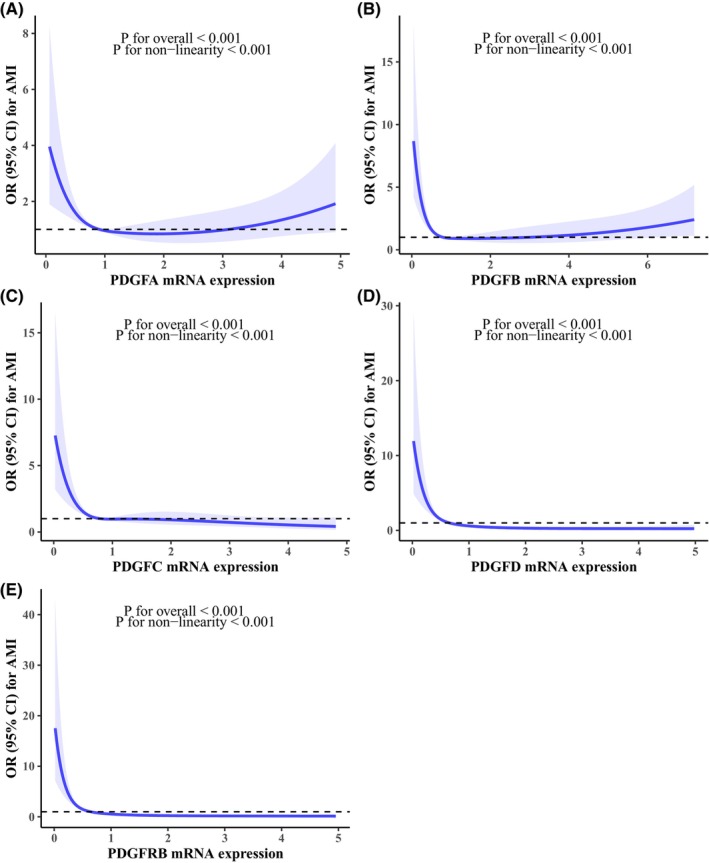
Dose–response relationship of PDGF/PDGFRB mRNA expression with AMI by restricted cubic spline regression. (A) restricted cubic spline regression of PDGFA mRNA expression and AMI; (B) restricted cubic spline regression of PDGFB mRNA expression and AMI; (C) restricted cubic spline regression of PDGFC mRNA expression and AMI; (D) restricted cubic spline regression of PDGFD mRNA expression and AMI; (E) restricted cubic spline regression of PDGFRB mRNA expression and AMI. ORs are indicated by solid lines and 95% CIs by shaded areas. Logistic regression models were adjusted for age, gender, smoking, drinking, hypertension, diabetes and dyslipidemia. AMI, acute myocardial infarction; CI, confidence interval; OR, odds ratio; PDGFA, platelet‐derived growth factor A; PDGFB, platelet‐derived growth factor B; PDGFC, platelet‐derived growth factor C; PDGFD, platelet‐derived growth factor D; PDGFRB, platelet‐derived growth factor receptor β.

Figure 3 shows the dose–response relationship of *PDGF/PDGFRB* mRNA expression with AMI by RCS regression. Non‐linear correlations were observed between five gene expressions and the risk of AMI, with “U”‐shaped curves for *PDGFA*, *PDGFB* and “L”‐shaped curves for *PDGFC*, *PDGFD* and *PDGFRB* (all *p* for non‐linearity <0.001). Based on results of RCS analyses, expression levels of five genes were divided into four groups (quartiles) with the high group (4th quartile) as reference. Logistic regression analyses indicated that low *PDGFA* and *PDGFB* expressions were associated with an increased risk of AMI (adjusted OR, 1.91 [95% CI, 1.15–3.18]; and OR, 3.63 [95% CI, 2.14–6.23]). Moreover, subjects with *PDGFC* expression levels in low and medium‐low groups had 3.95‐ and 1.69‐fold risk for AMI when compared to those with high *PDGFC* expressions (adjusted OR, 3.95 [95% CI, 2.33–6.79]; and OR, 1.69 [95% CI, 1.02–2.80]). In addition, the low, medium‐low, and medium‐high groups of *PDGFD* and *PDGFRB* expressions were all related with a higher risk of AMI compared to the high group. Adjusted ORs were 18.09 (95% CI, 9.81–34.60), 5.06 (95% CI, 2.92–8.94), 2.21 (95% CI, 1.27–3.89) for *PDGFD*; and 27.78 (95% CI, 14.39–56.23), 8.19 (95% CI, 4.61–14.96), and 2.33 (95% CI, 1.31–4.23) for *PDGFRB* (Table S11).

### 3.6 *PDGF* / *PDGFRB* expressions by SNP genotypes and wGRS‐mRNA correlation

**FIGURE 4:**
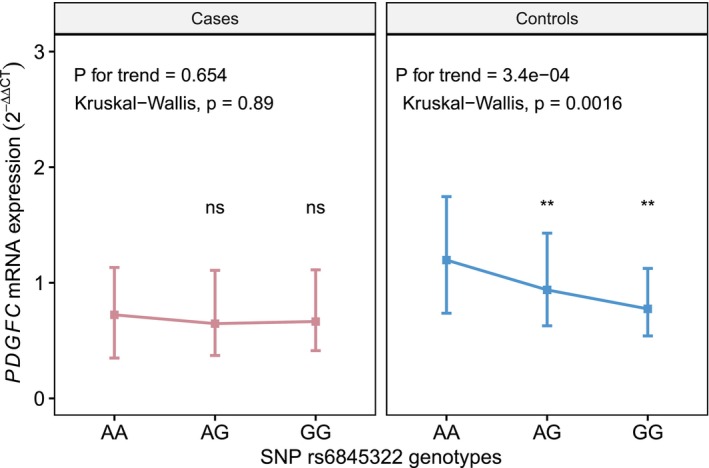
PDGFC mRNA expression levels by SNP rs6845322 genotypes. Line plots with median points and quartile error bars. Kruskal–Wallis test was used for the comparison among genotypes. Jonckheere–Terpstra test was used for the trend analysis. **: Significant at 0.01 level, *p* < 0.01. ns, Not significant, *p* > 0.05. PDGFC, platelet‐derived growth factor C; SNP, single nucleotide polymorphism.

We also compared the mRNA expression levels of each gene among three genotypes of the corresponding pathway SNPs (Table S12). It was found that *PDGFC* mRNA levels significantly differed across the AA, AG and GG genotypes of *PDGFC* variant rs6845322 in the control group (*p* = 0.002), and the expression showed a decreasing trend as the number of the mutant allele increased (*p* for trend = 3.4 × 10^−4^) (Figure 4). Table S13 displays the expression quantitative trait loci (eQTL) results of 12 SNPs and PDGFs/PDGFRB expressions. Based on the common public eQTL database, 12 SNPs in this study except rs13053714 are recorded to be cis‐eQTL, which have significant associations with their corresponding nearby genes. Figure S2 shows the scatterplot and correlation curves between wGRS and *PDGF/PDGFRB* mRNA expression levels. As indicated by the Spearman coefficients and *p* values, there were no significant correlations between wGRS and *PDGF/PDGFRB* mRNA levels in controls, cases at admission and at discharge (all *p* > 0.05).

### 3.7 Discrimination of wGRS, *PDGF* / *PDGFRB* mRNAs and traditional risk factors for CAD

**FIGURE 5:**
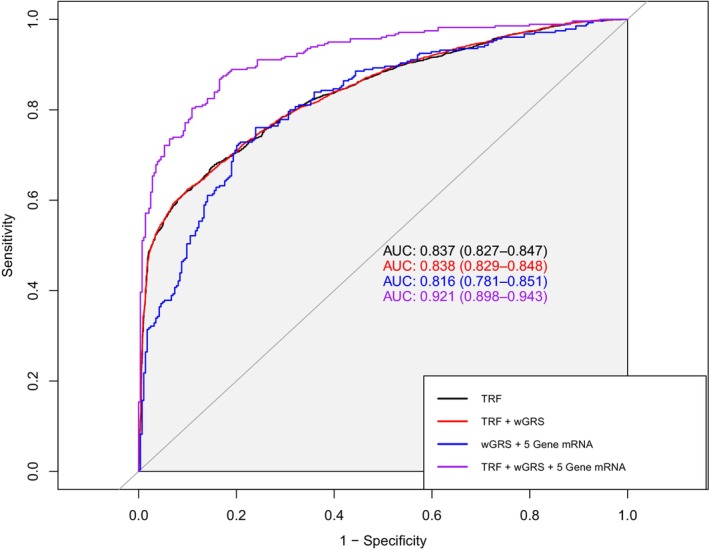
Receiver operating characteristic curves of wGRS, PDGF/PDGFRB mRNAs and traditional risk factors for coronary artery disease. Traditional risk factors included age, gender, smoking, drinking, hypertension, diabetes and dyslipidemia. Five genes were PDGFA, PDGFB, PDGFC, PDGFD and PDGFRB. AUC, area under the curve; TRF, traditional risk factors; wGRS, weighted genetic risk score.

We further evaluated the discrimination performance of wGRS and *PDGF/PDGFRB* mRNAs for CAD and made comparisons with TRF (Table S14). The TRF model consisted of age, gender, smoking, drinking, hypertension, diabetes and dyslipidemia, which had an AUC of 0.837 (95% CI, 0.827–0.847). Adding wGRS to the traditional risk model only led to a slight increment for the AUC (difference, 0.1%; *p* = 0.117). However, when we added the single gene mRNA of *PDGFC*, *PDGFD*, or *PDGFRB* to wGRS and TRF, the AUC was significantly improved to 0.880 (95% CI, 0.852–0.908; *p* = 0.032), 0.890 (95% CI, 0.863–0.917; *p* = 0.001), and 0.919 (95% CI, 0.896–0.942; *p* < 0.001), respectively. Furthermore, the combined model of TRF, wGRS, and five gene mRNAs had the best discriminative ability for CAD with an AUC of 0.921 (95% CI, 0.898–0.943; *p* < 0.001) compared to any other models. ROC curves of four main models of wGRS, *PDGF/PDGFRB* mRNAs and TRF were plotted in Figure 5.

## 4 DISCUSSION

In this case–control study, we investigated the association of genetic variations and mRNA expressions of PDGF/PDGFRB pathway genes with CAD in the Chinese population. The wGRS, constructed with 13 SNPs, had a positive linear relationship with the risk of CAD in both unadjusted and adjusted models, and the medium and high wGRS had a higher risk of CAD compared to the low wGRS group. Additionally, we observed that *PDGF/PDGFRB* mRNA expressions were all down‐regulated in AMI patients. There were non‐linear correlations between five gene expressions and the risk of AMI with “U”‐shaped curves for *PDGFA*, *PDGFB* and “L”‐shaped curves for *PDGFC*, *PDGFD* and *PDGFRB*. Our results also demonstrated that adding wGRS and five gene mRNAs to the traditional risk model significantly improved the discrimination for CAD.

GWAS have identified hundreds of susceptibility loci that contribute to the genetic risk for CAD, yet the contribution of these genome‐wide significant variants to CAD heritability is limited^32^ and over 90% variants fall in non‐coding areas of human genome.^33^ Updated evidence support the proposition that much of the genetic risk for complex diseases might also be attributed to rare variants with large effects or joint contributions from common variants with small effects.^15^ In this study, we were first to find that genetic variation A >G of SNP rs246390 at *PDGFRB* made a difference in CAD susceptibility, which was never reported in GWAS or any other research by far. Besides this single variant at *PDGFRB*, we only observed very weak effects of other 12 PDGF/PDGFRB pathway SNPs on CAD in our population. However, when we combined all 13 SNPs to construct a wGRS, the wGRS exhibited a significant effect on the increased risk of CAD with a positive linear trend. By focusing on variations in a biological pathway, our study placed a set of weakly associated variants into a broader and clearer functional context for better understanding how genes of these loci regulate molecular processes underling CAD risk. Genetic variants are valuable biomarkers because they are quantifiable and stable long time before the onset of disease, but GRS or polygenic risk score is a more comprehensive tool to assess individuals' inherited risk for complex disease like CAD. Previous studies have shown that GRS developed with GWAS variants or polymorphisms in biological pathways could aid in the risk stratification and identifying high‐risk population, as well as predicting CAD prospectively.^34^, ^35^, ^36^

Additionally, we found that apart from SNP rs246390, rs5757573 and rs13053714 at *PDGFB*, rs342309 at *PDGFC*, rs3828610 and rs9324641 at *PDGFRB* were also risk variants for CAD in certain subgroups of the study population. Our results indicated that the genetic influence of PDGF/PDGFRB pathway genes on CAD may not independent of environmental factors. Future research could focus on the interaction of these variations and traditional risk factors of CAD. For those risk allele carriers in specific high‐risk sub‐population, individualized controls for modifiable risk factors should be paid more attention.

mRNA reflects the expression and regulation of genetic information in specific disease states. Transcriptome‐level research or gene expression profiling is a promising approach for identification of disease‐related molecular biomarkers and exploration of how genetic variations involving in gene expressing regulation. In our study, the mRNA expression of *PDGF/PDGFRB* genes in peripheral blood leukocytes were down‐regulated in AMI patients just admitted into hospital compared to controls and elevated as the patients discharged, with *PDGFRB* expression having the maximum change. The mRNA expressions of *PDGF/PDGFRB* had significant non‐linear correlations with the risk of AMI, with “U”‐shaped trend for *PDGFA*, *PDGFB* and “L”‐shaped trend for *PDGFC*, *PDGFD* and *PDGFRB*. Our results accord with another study which verified that the mRNA expression of *PDGFRB* in peripheral blood mononuclear cells was lower among CAD patients than controls.^37^ Similarly, our findings could also be supported by a previous finding of lower plasma PDGF levels in patients with unstable angina pectoris and acute myocardial infarction than normal control subjects.^38^ However, there is also research detecting opposite changes in the expression and proteins of PDGFs and PDGFRs in different regions of the infarcted rat heart or during different stages of myocardial infarction.^23^ They found that the expression of *PDGFA*, *PDGFD* and *PDGFRB* in the infarcted myocardium were increased after myocardial infarction, whereas *PDGFB* and *PDGFC* mRNAs were reduced and protein levels at infarcted myocardium were all significantly reduced in the early stage of myocardial infarction. Existing evidence suggest a multifaceted role of PDGF/PDGFR pathway in the development and progression of CAD with either beneficial or adverse effects on atherosclerosis.^9^, ^39^, ^40^, ^41^ The exact mechanism of how PDGF/PDGFR signalling regulation contributing to CAD pathogenesis needs further investigation.

Of note, our current results indicated that wGRS of 13 SNP in the PDGF/PDGFR pathway could only make a slight increment for the AUC, while the combined model of five gene mRNAs, wGRS and TRF significantly improved the discrimination for CAD. These results reflect the limitation of genetic variations for CAD diagnosis or prediction,^42^ and suggest that differentially expressed PDGF/PDGFRB mRNAs might be useful biomarkers in assisting CAD discrimination.

This is the first study to focus on the association of PDGF/PDGFRB pathway with CAD in the population level by integrating genomic and transcriptomic data. The relatively large sample size and high‐quality experiments guarantee the accuracy and reliability of our results. However, some limitations should also be taken into consideration. First, since we were not able to detect the *PDGFRA* mRNA expressing in peripheral leukocytes, we did not include PDGFA in our study and no SNPs at *PDGFRA* were selected. The *PDGFRA* mRNA was more likely to be expressed in tissue cells. Unfortunately, this study focused on *PDGFs*/*PDGFRB* mRNA expressions in peripheral blood leukocytes and did not evaluate the expression in other cell types or other tissues such as vascular smooth muscle cells in myocardium, so the celltype‐specific gene expressions need further evaluation. Second, we only selected tagSNPs at the PDGF/PDGFRB pathway genes with MAF ≥0.05; thus, some rare variants with large effects but MAF <0.05 might be missed. Third, the protein levels of PDGFs and PDGFRB were not measured in this study, which would limit the comprehension for the functional mechanism of association between PDGFs and CAD. Fourth, we were not able to evaluate the impact of medication use on *PDGF* expression levels because of limited information. Furthermore, CAD patients in this research were recruited from a single hospital and all participants came from northern China with Han ancestry, so the potential selection bias was unavoidable and representativeness was lacking. Finally, the case–control study design was unable to infer a causal relationship between *PDGF*/*PDGFRB* mRNA expressions and CAD. Hence, the prospective cohort study or nested case–control study with larger sample sizes and diverse nationalities are warranted to validate our findings.

## 5 CONCLUSIONS

In conclusion, this research provides important new insights into the role of PDGF/PDGFRB signalling pathway in the molecular process of CAD occurrence with genomic and transcriptomic evidence. Genetic variations in the PDGF/PDGFRB pathway contribute to CAD susceptibility with a significantly joint effect in Chinese population. These findings may aid in further characterizing the genetic basis of CAD and bring us closer to precision medicine approaches for CAD. Additionally, the down‐regulated mRNA expression of PDGF/PDGFRB genes in peripheral leukocytes were found to be highly correlated with an increased risk of AMI, which might as well offer clues for the potential of PDGF/PDGFRB mRNAs as blood‐based biomarkers for AMI. Furthermore, our findings also shed light on the discriminative value of variants and mRNAs of PDGF/PDGFRB pathway genes for CAD, and highlight the importance to deeply explore the participation of PDGF/PDGFRB signalling pathway in the molecular mechanism of CAD.

## AUTHOR CONTRIBUTIONS

**Pengfei Wei:** Formal analysis (equal); methodology (supporting); resources (lead); validation (lead); writing – review and editing (supporting). **Hankun Xie:** Formal analysis (equal); software (lead); visualization (lead); writing – original draft (lead); writing – review and editing (lead). **Junxiang Sun:** Investigation (lead); resources (lead). **Qian Zhuang:** Investigation (supporting); resources (supporting). **Jichao Xie:** Investigation (supporting); resources (supporting). **Yunjie Yin:** Investigation (supporting); resources (supporting). **Fangyuan Liu:** Investigation (lead). **Wen Li:** Investigation (supporting). **Changying Chen:** Investigation (supporting). **Feifan Wang:** Investigation (supporting). **Xu Han:** Investigation (supporting). **Liang Xu:** Supervision (supporting). **Xianghai Zhao:** Supervision (supporting). **Yanchun Chen:** Supervision (supporting). **Song Yang:** Funding acquisition (lead); project administration (equal). **Chong Shen:** Conceptualization (lead); methodology (equal); project administration (equal).

## FUNDING INFORMATION

This study was supported by grants from the National Natural Science Foundation of China (82173611, 81872686), Scientific Research Project of Yixing Municipal Health Commission (YWKJ202207), and Priority Academic Program Development of Jiangsu Higher Education Institutions (Public Health and Preventive Medicine).

## CONFLICT OF INTEREST STATEMENT

The authors declare that they have no conflicts of interest.

## Supporting information


Data S1.

## Data Availability

The data that support the findings of this study are available from the corresponding author upon reasonable request.
