# Supplementary material for: Associations of genetic variation and mRNA expression of PDGF/PDGFRB pathway genes with coronary artery disease in the Chinese population
Source: J Cell Mol Med. 2024 Nov 21;28(22):e70193. doi: 10.1111/jcmm.70193 (PMC11579943; doi:10.1111/jcmm.70193)
Supplement: Supplementary file 1 — Data S1. [file JCMM-28-e70193-s001.docx]

**Table S1. Genetic information and functional prediction of 13 tagSNPs in the PDGF/PDGFRB pathway**

| **No** | **SNP** | **Chr:Position** | **Allele** | **Nearby Gene** | **Enhancer** | **TFBS** | **eQTL** | **MAF** | **Genotype Frequency** | **Allele Frequency** | **Ne** | **PIC** | ***P**** | ***P***^†^ |
| --- | --- | --- | --- | --- | --- | --- | --- | --- | --- | --- | --- | --- | --- | --- |
| 1 | rs28472363 | 7:551122 | G/A | *PDGFA* | - | - | Y | 0.388 | 0.455(GG), 0.435(GA), 0.110(AA) | 0.673(G), 0.328(A) | 1.791 | 0.344 | 0.304 | 0.968 |
| 2 | rs5757573 | 22:39633622 | T/C | *PDGFB* | - | - | Y | 0.087 | 0.869(TT), 0.125(TC), 0.006(CC) | 0.932(T), 0.069(C) | 1.134 | 0.111 | 0.113 | 0.190 |
| 3 | rs13053714 | 22:39626572 | G/A | *PDGFB* | - | - | - | 0.117 | 0.754(GG), 0.230(GA), 0.015(AA) | 0.869(G), 0.130(A) | 1.315 | 0.211 | 0.275 | 0.334 |
| 4 | rs1834389 | 4:157718612 | A/C | *PDGFC* | Y | - | Y | 0.165 | 0.711(AA), 0.263(AC), 0.027(CC) | 0.843(A), 0.159(C) | 1.373 | 0.235 | 0.347 | 0.584 |
| 5 | rs342309 | 4:157811441 | G/A | *PDGFC* | Y | - | Y | 0.257 | 0.531(GG), 0.393(GA), 0.076(AA) | 0.728(G), 0.273(A) | 1.688 | 0.325 | 0.491 | 0.120 |
| 6 | rs6845322 | 4:157884105 | A/G | *PDGFC* | Y | - | Y | 0.471 | 0.316(AA), 0.500(AG), 0.184(GG) | 0.566(A), 0.434(G) | 1.965 | 0.370 | 0.150 | 0.754 |
| 7 | rs1053861 | 11:103778154 | C/T | *PDGFD* | - | - | Y | 0.486 | 0.302(CC), 0.497(CT), 0.200(TT) | 0.551(C), 0.449(T) | 1.983 | 0.373 | 0.724 | 0.773 |
| 8 | rs11226185 | 11:103991641 | T/C | *PDGFD* | Y | - | Y | 0.290 | 0.487(TT), 0.426(TC), 0.087(CC) | 0.700(T), 0.300(C) | 1.729 | 0.333 | 0.268 | 0.802 |
| 9 | rs4755010 | 11:104034148 | G/C | *PDGFD* | Y | Y | Y | 0.276 | 0.507(GG), 0.417(GC), 0.076(CC) | 0.716(G), 0.285(C) | 1.678 | 0.322 | 0.049 | 0.114 |
| 10 | rs6579775 | 5:149533848 | C/T | *PDGFRB* | Y | - | Y | 0.219 | 0.683(CC), 0.289(CT), 0.028(TT) | 0.828(C), 0.173(T) | 1.403 | 0.246 | 0.331 | 0.801 |
| 11 | rs3828610 | 5:149535625 | C/A | *PDGFRB\|\|CDX1* | Y | Y | Y | 0.417 | 0.337(CC), 0.484(CA), 0.179(AA) | 0.579(C), 0.421(A) | 1.962 | 0.370 | 0.575 | 0.774 |
| 12 | rs246390 | 5:149496321 | A/G | *PDGFRB* | - | Y | Y | 0.350 | 0.425(AA), 0.442(AG), 0.134(GG) | 0.646(A), 0.355(G) | 1.808 | 0.347 | 0.006 | 0.106 |
| 13 | rs9324641 | 5:149527844 | C/T | *PDGFRB* | Y | - | Y | 0.422 | 0.346(CC), 0.489(CT), 0.165(TT) | 0.591(C), 0.410(T) | 1.940 | 0.367 | 0.345 | 0.279 |

* *P* value of the Hardy-Weinberg equilibrium test in the total population.

† *P* value of the Hardy-Weinberg equilibrium test in the control population.

Abbreviations: Chr, chromosome; eQTL, expression quantitative trait loci; MAF, minor allele frequency; Ne, effective number of alleles; PDGFA, platelet-derived growth factor A; PDGFB, platelet-derived growth factor B; PDGFC, platelet-derived growth factor C; PDGFD, platelet-derived growth factor D; PDGFRB, platelet-derived growth factor receptor β; PIC, polymorphism information content; SNP, single nucleotide polymorphism; TFBS, transcription factor binding site.

**Table S2. Taqman primers and probes for genotyping**

| **SNP** | **Primers (5'-3')** | **Probes (5'-3')** |
| --- | --- | --- |
| rs28472363 | F: CTGTGGGGTCTGCATTTTGC | G: TCAGACCACTCTTGA |
|  | R: CAGTGAATCCTACAGCACTTGC | A: TTCAGACCACTTTTGA |
| rs5757573 | F: CGCCCTCAAGCAAGTTCATTC | T: TGGAGAGCACCGAAG |
|  | R: TCTGTGGGTTGGGTACTTTGTC | C: TGGAGAGCACCGGAG |
| rs13053714 | CGGTATCCTCATTCCCGGA | G; ACCATCTCCCGCCT |
|  | CCCTCCTAGATGAAGCCTCC | A: AACCATCTCCTGCCT |
| rs1834389 | F: CAGAAAAAGGAAAGTGATACGCAAA | A: AGTGAGGTACAGGAAC |
|  | R: CATGTTCAAATGCAGAGCTGTGA | C: TGAGGTACAGAAACAG |
| rs342309 | F: TCAGACTCACAAGACCTCAAAGCT | G: AACATGCTGTTTTTTTGTAGTG |
|  | R: AGAAAGTGGCAAGCAGAATGG | A: TGTTTTTTTGCAGTGGAC |
| rs6845322 | F: CATCCAACTACCCTCTAAAATGCAT | A: CTGCTCCCGGGTC |
|  | R: TGCAAGGATGTGGATTTACATTTC | G: CTGCTGCTCCCAGGT |
| rs1053861 | F: TTGCACTTCTAACATGTAGCCAATAA | C: AATAAAAAGCTGGCCGTACA |
|  | R: AATGAGCCATGCGGAAATG | T: AAAAAGCTGGCCATACA |
| rs11226185 | F: TGTACATGCCCTACTCCCAAAGT | T: ATATTTGGCTTTGTTCTC |
|  | R: GTGGGCCTGCTACGTAAAAGA | C: TTTGGCTCTGTTCTC |
| rs4755010 | F: CGATTTCTCCTCTCACTCGTAAAAA | G: TCTTGAGTCCTTCCAC |
|  | R: GCCCCTTGGTCAGACTCAAC | C: TACTCTTGACTCCTTCCA |
| rs6579775 | F: GCAGGATGGTGCGCATTC | C: TCTGGGAATTTTTGG |
|  | R: TGCCCCATTACTCCAAACTTG | T: TTCTGGGAATTCTTGGG |
| rs3828610 | F: CGTGGGCTGTGGAATTTCTAA | C: AGTAAGCAGAGGGAAAA |
|  | R: GTGCGTCTGTTTTCAATTTCAGTT | A: AGTAAGCAGAGTGAAA |
| rs246390 | F: GCATTCACACCTCAAGCTGTGT | A: CACTCCTCTGCGCCA |
|  | R: AAGAGGCCAACCATCCAAGAGT | G: TCTGCACCATCCAC |
| rs9324641 | F: GTAAGCGTTTACTGAGCACCTACTGT | C: CCATATTAGGAAGTTGAAG |
|  | R: CAGGATACCTACCTAACAACGATCTG | T: CCATATTAGGAAGTTGGAG |

**Table S3. mRNA primer sequences of *PDGFs*/*PDGFRB* and reference genes for qPCR assay**

| **Gene** | **Primer Sequences (5' to 3')** |
| --- | --- |
| *PDGFA* | Forward: GCAAGACCAGGACGGTCATTT |
|  | Reverse: GGCACTTGACACTGCTCGT |
| *PDGFB* | Forward: CTCGATCCGCTCCTTTGATGA |
|  | Reverse: CGTTGGTGCGGTCTATGAG |
| *PDGFC* | Forward: ATTTGGGCTTGAAGACCCAGA |
|  | Reverse: CCAGCGCCCTAATATAGTTCCA |
| *PDGFD* | Forward: TTGTACCGAAGAGATGAGACCA |
|  | Reverse: GCTGTATCCGTGTATTCTCCTGA |
| *PDGFRB* | Forward: TGATGCCGAGGAACTATTCATCT |
|  | Reverse: TTTCTTCTCGTGCAGTGTCAC |
| *GAPDH* | Forward: GACAGTCAGCCGCATCTTCT |
|  | Reverse: TTAAAAGCAGCCCTGGTGAC |

Abbreviations: GAPDH, glyceraldehyde-3-phosphate dehydrogenase; qPCR, quantitative polymerase chain reaction.

**Table S4. Association analyses of** **PDGF/PDGFRB pathway SNPs and coronary artery disease**

| **Gene** | **SNP** | **Additive Model** | | | **Dominant Model** | | **Recessive Model** | |
| --- | --- | --- | --- | --- | --- | --- | --- | --- |
|  |  | **OR (95% CI)** | ***P* value** | ***P**** | **OR (95% CI)** | ***P* value** | **OR (95% CI)** | ***P* value** |
| *PDGFA* | rs28472363 | 0.990 (0.902-1.085) | 0.823 | 0.488 | 0.965 (0.853-1.093) | 0.577 | 1.042 (0.856-1.269) | 0.680 |
| *PDGFB* | rs5757573 | 0.860 (0.725-1.020) | 0.084 | 0.163 | 0.866 (0.723-1.039) | 0.122 | 0.551 (0.228-1.333) | 0.186 |
| *PDGFB* | rs13053714 | 1.092 (0.958-1.244) | 0.188 | 0.187 | 1.134 (0.982-1.310) | 0.086 | 0.784 (0.468-1.313) | 0.355 |
| *PDGFC* | rs1834389 | 1.086 (0.965-1.223) | 0.173 | 0.185 | 1.090 (0.952-1.249) | 0.213 | 1.187 (0.812-1.735) | 0.375 |
| *PDGFC* | rs342309 | 1.087 (0.985-1.198) | 0.096 | 0.167 | 1.137 (1.005-1.287) | 0.041 | 1.013 (0.799-1.284) | 0.916 |
| *PDGFC* | rs6845322 | 0.951 (0.870-1.039) | 0.269 | 0.237 | 0.979 (0.857-1.117) | 0.749 | 0.877 (0.747-1.030) | 0.109 |
| *PDGFD* | rs1053861 | 1.064 (0.975-1.162) | 0.161 | 0.183 | 1.128 (0.987-1.291) | 0.078 | 1.034 (0.887-1.206) | 0.667 |
| *PDGFD* | rs11226185 | 1.043 (0.948-1.148) | 0.387 | 0.309 | 1.050 (0.928-1.188) | 0.440 | 1.069 (0.861-1.328) | 0.545 |
| *PDGFD* | rs4755010 | 0.986 (0.894-1.087) | 0.772 | 0.472 | 0.961 (0.850-1.087) | 0.531 | 1.060 (0.841-1.337) | 0.621 |
| *PDGFRB* | rs6579775 | 1.039 (0.926-1.166) | 0.515 | 0.373 | 1.068 (0.936-1.219) | 0.330 | 0.890 (0.616-1.286) | 0.535 |
| *PDGFRB* | rs3828610 | 1.087 (0.995-1.187) | 0.064 | 0.154 | 1.119 (0.982-1.276) | 0.092 | 1.112 (0.947-1.306) | 0.195 |
| *PDGFRB* | rs246390 | **0.887 (0.810-0.970)** | **0.009** | 0.063 | 0.873 (0.770-0.988) | 0.032 | 0.815 (0.678-0.980) | 0.030 |
| *PDGFRB* | rs9324641 | 1.018 (0.931-1.114) | 0.690 | 0.444 | 1.004 (0.882-1.143) | 0.956 | 1.059 (0.897-1.250) | 0.501 |

*: false discovery rate adjustment.

Additive model: wild type vs heterozygote type vs mutant type.

Dominant model: wild type vs heterozygote type/mutant type.

Recessive model: wild type/heterozygote type vs mutant type.

All models were adjusted for age, gender, smoking, drinking, hypertension, diabetes, and dyslipidemia.

Abbreviations: CI, confidence interval; OR, odds ratio; SNP, single nucleotide polymorphism.

**Table S5. Association analyses of PDGF/PDGFRB pathway SNPs and coronary artery disease by age and gender**

| **Gene** | **SNP** | **Subgroups** | **Additive Model** | | **Dominant Model** | | **Recessive Model** | |
| --- | --- | --- | --- | --- | --- | --- | --- | --- |
|  |  |  | **OR (95% CI)** | ***P* value** | **OR (95% CI)** | ***P* value** | **OR (95% CI)** | ***P* value** |
| *PDGFA* | rs28472363 | <65 years | 0.98 (0.86-1.12) | 0.765 | 0.94 (0.79-1.13) | 0.529 | 1.05 (0.79-1.40) | 0.714 |
|  |  | ≥65 years | 0.99 (0.87-1.13) | 0.881 | 0.98 (0.82-1.16) | 0.780 | 1.02 (0.77-1.34) | 0.901 |
|  |  | Male | 0.95 (0.85-1.06) | 0.384 | 0.90 (0.78-1.05) | 0.180 | 1.03 (0.82-1.30) | 0.782 |
|  |  | Female | 1.09 (0.92-1.30) | 0.328 | 1.14 (0.90-1.45) | 0.263 | 1.06 (0.73-1.54) | 0.760 |
| *PDGFB* | rs5757573 | <65 years | 0.80 (0.62-1.03) | 0.087 | 0.83 (0.63-1.08) | 0.157 | 0.23 (0.05-1.02) | 0.054 |
|  |  | ≥65 years | 0.93 (0.73-1.17) | 0.529 | 0.92 (0.72-1.19) | 0.535 | 0.88 (0.29-2.64) | 0.822 |
|  |  | Male | **0.80 (0.65-0.98)** | **0.030** | 0.80 (0.64-0.99) | 0.043 | 0.50 (0.17-1.46) | 0.206 |
|  |  | Female | 1.04 (0.77-1.41) | 0.802 | 1.06 (0.77-1.47) | 0.713 | 0.68 (0.14-3.34) | 0.635 |
|  | rs13053714 | <65 years | 1.11 (0.92-1.34) | 0.261 | 1.16 (0.94-1.42) | 0.169 | 0.84 (0.41-1.74) | 0.641 |
|  |  | ≥65 years | 1.07 (0.89-1.28) | 0.500 | 1.11 (0.91-1.36) | 0.315 | 0.71 (0.34-1.48) | 0.358 |
|  |  | Male | 1.01 (0.87-1.18) | 0.859 | 1.06 (0.90-1.26) | 0.480 | 0.60 (0.33-1.10) | 0.097 |
|  |  | Female | **1.32 (1.03-1.69)** | **0.028** | 1.32 (1.01-1.72) | 0.042 | 2.08 (0.72-5.97) | 0.176 |
| *PDGFC* | rs1834389 | <65 years | 1.10 (0.92-1.30) | 0.293 | 1.14 (0.94-1.39) | 0.179 | 0.91 (0.54-1.56) | 0.739 |
|  |  | ≥65 years | 1.08 (0.91-1.27) | 0.392 | 1.04 (0.86-1.26) | 0.671 | 1.55 (0.90-2.65) | 0.111 |
|  |  | Male | 1.10 (0.96-1.27) | 0.166 | 1.11 (0.95-1.30) | 0.194 | 1.20 (0.77-1.86) | 0.420 |
|  |  | Female | 1.04 (0.82-1.31) | 0.748 | 1.04 (0.80-1.35) | 0.793 | 1.12 (0.53-2.36) | 0.765 |
|  | rs342309 | <65 years | 1.15 (1.00-1.33) | 0.046 | 1.25 (1.05-1.49) | 0.013 | 1.02 (0.73-1.43) | 0.924 |
|  |  | ≥65 years | 1.03 (0.89-1.18) | 0.709 | 1.04 (0.87-1.23) | 0.684 | 1.02 (0.73-1.42) | 0.905 |
|  |  | Male | **1.14 (1.02-1.28)** | **0.026** | 1.21 (1.04-1.40) | 0.011 | 1.08 (0.82-1.42) | 0.581 |
|  |  | Female | 0.96 (0.80-1.16) | 0.688 | 0.99 (0.78-1.25) | 0.920 | 0.82 (0.51-1.33) | 0.427 |
|  | rs6845322 | <65 years | 0.96 (0.84-1.09) | 0.508 | 1.02 (0.85-1.24) | 0.806 | 0.84 (0.67-1.06) | 0.138 |
|  |  | ≥65 years | 0.94 (0.83-1.07) | 0.358 | 0.93 (0.77-1.12) | 0.427 | 0.92 (0.74-1.16) | 0.488 |
|  |  | Male | 0.99 (0.89-1.10) | 0.869 | 1.01 (0.87-1.19) | 0.859 | 0.95 (0.79-1.15) | 0.614 |
|  |  | Female | 0.86 (0.72-1.02) | 0.074 | 0.91 (0.71-1.16) | 0.435 | 0.69 (0.50-0.95) | 0.022 |
| *PDGFD* | rs1053861 | <65 years | 1.03 (0.91-1.17) | 0.600 | 1.06 (0.87-1.28) | 0.575 | 1.03 (0.83-1.29) | 0.784 |
|  |  | ≥65 years | 1.09 (0.97-1.24) | 0.148 | 1.20 (1.00-1.45) | 0.056 | 1.04 (0.84-1.29) | 0.726 |
|  |  | Male | 1.02 (0.92-1.14) | 0.639 | 1.04 (0.89-1.22) | 0.599 | 1.02 (0.85-1.22) | 0.826 |
|  |  | Female | 1.17 (0.99-1.38) | 0.066 | 1.39 (1.07-1.79) | 0.014 | 1.06 (0.79-1.42) | 0.698 |
|  | rs11226185 | <65 years | 1.00 (0.87-1.15) | 0.998 | 1.01 (0.84-1.20) | 0.941 | 0.98 (0.71-1.35) | 0.889 |
|  |  | ≥65 years | 1.08 (0.94-1.23) | 0.269 | 1.08 (0.91-1.29) | 0.361 | 1.15 (0.85-1.54) | 0.362 |
|  |  | Male | 1.03 (0.92-1.16) | 0.556 | 1.03 (0.89-1.20) | 0.656 | 1.08 (0.83-1.39) | 0.580 |
|  |  | Female | 1.07 (0.89-1.28) | 0.478 | 1.09 (0.86-1.37) | 0.480 | 1.08 (0.72-1.61) | 0.713 |
|  | rs4755010 | <65 years | 1.11 (0.96-1.28) | 0.148 | 1.16 (0.97-1.38) | 0.108 | 1.07 (0.76-1.51) | 0.710 |
|  |  | ≥65 years | 0.89 (0.78-1.02) | 0.099 | 0.81 (0.69-0.97) | 0.020 | 1.06 (0.78-1.46) | 0.696 |
|  |  | Male | 1.04 (0.93-1.17) | 0.464 | 1.03 (0.89-1.19) | 0.676 | 1.14 (0.87-1.49) | 0.342 |
|  |  | Female | 0.85 (0.70-1.02) | 0.082 | 0.80 (0.63-1.01) | 0.065 | 0.86 (0.54-1.36) | 0.511 |
| *PDGFRB* | rs6579775 | <65 years | 0.99 (0.84-1.17) | 0.941 | 1.00 (0.82-1.20) | 0.968 | 0.97 (0.56-1.66) | 0.898 |
|  |  | ≥65 years | 1.09 (0.93-1.28) | 0.303 | 1.15 (0.95-1.38) | 0.151 | 0.84 (0.51-1.40) | 0.512 |
|  |  | Male | 1.10 (0.96-1.26) | 0.171 | 1.16 (0.99-1.36) | 0.060 | 0.83 (0.54-1.28) | 0.405 |
|  |  | Female | 0.91 (0.73-1.13) | 0.383 | 0.87 (0.68-1.12) | 0.289 | 1.07 (0.53-2.18) | 0.850 |
|  | rs3828610 | <65 years | 1.14 (1.00-1.29) | 0.043 | 1.17 (0.97-1.41) | 0.098 | 1.22 (0.97-1.53) | 0.096 |
|  |  | ≥65 years | 1.04 (0.92-1.18) | 0.518 | 1.07 (0.89-1.29) | 0.461 | 1.03 (0.82-1.29) | 0.786 |
|  |  | Male | 1.07 (0.97-1.19) | 0.181 | 1.08 (0.93-1.26) | 0.317 | 1.12 (0.93-1.36) | 0.226 |
|  |  | Female | 1.11 (0.94-1.32) | 0.216 | 1.21 (0.94-1.56) | 0.137 | 1.07 (0.79-1.45) | 0.677 |
|  | rs246390 | <65 years | **0.87 (0.76-0.99)** | **0.030** | 0.83 (0.69-0.99) | 0.042 | 0.83 (0.64-1.07) | 0.149 |
|  |  | ≥65 years | 0.91 (0.80-1.03) | 0.137 | 0.92 (0.77-1.09) | 0.345 | 0.80 (0.62-1.04) | 0.102 |
|  |  | Male | 0.92 (0.82-1.02) | 0.101 | 0.91 (0.78-1.05) | 0.204 | 0.85 (0.69-1.05) | 0.141 |
|  |  | Female | **0.82 (0.69-0.98)** | **0.029** | 0.80 (0.63-1.01) | 0.057 | 0.74 (0.52-1.06) | 0.101 |
|  | rs9324641 | <65 years | 1.09 (0.96-1.24) | 0.165 | 1.08 (0.89-1.29) | 0.435 | 1.22 (0.96-1.54) | 0.110 |
|  |  | ≥65 years | 0.96 (0.84-1.08) | 0.479 | 0.94 (0.79-1.13) | 0.534 | 0.94 (0.74-1.19) | 0.604 |
|  |  | Male | 1.01 (0.91-1.12) | 0.864 | 0.97 (0.84-1.13) | 0.726 | 1.08 (0.89-1.31) | 0.441 |
|  |  | Female | 1.04 (0.87-1.23) | 0.687 | 1.08 (0.84-1.39) | 0.546 | 1.00 (0.72-1.37) | 0.979 |

Age subgroup analyses were adjusted for age, gender, smoking, drinking, hypertension, diabetes, and dyslipidemia.

Gender subgroup analyses were adjusted for age, smoking, drinking, hypertension, diabetes, and dyslipidemia.

Abbreviations: CI, confidence interval; OR, odds ratio; SNP, single nucleotide polymorphism.

**Table S6. Association analyses of PDGF/PDGFRB pathway SNPs and coronary artery disease by smoking and drinking statuses**

| **Gene** | **SNP** | **Subgroups** | **Additive Model** | | **Dominant Model** | | **Recessive Model** | |
| --- | --- | --- | --- | --- | --- | --- | --- | --- |
|  |  |  | **OR (95% CI)** | ***P* value** | **OR (95% CI)** | ***P* value** | **OR (95% CI)** | ***P* value** |
| *PDGFA* | rs28472363 | Smoking | 0.90 (0.77-1.04) | 0.148 | 0.85 (0.70-1.04) | 0.116 | 0.91 (0.66-1.26) | 0.568 |
|  |  | Non-smoking | 1.06 (0.94-1.19) | 0.338 | 1.06 (0.90-1.25) | 0.461 | 1.12 (0.87-1.44) | 0.383 |
|  |  | Drinking | 0.86 (0.71-1.04) | 0.113 | 0.76 (0.59-0.97) | 0.030 | 1.02 (0.68-1.52) | 0.939 |
|  |  | Non-drinking | 1.04 (0.93-1.15) | 0.506 | 1.05 (0.91-1.21) | 0.499 | 1.04 (0.83-1.31) | 0.733 |
| *PDGFB* | rs5757573 | Smoking | **0.72 (0.54-0.95)** | **0.021** | 0.74 (0.55-0.99) | 0.046 | 0.18 (0.04-0.91) | 0.038 |
|  |  | Non-smoking | 0.96 (0.78-1.20) | 0.745 | 0.96 (0.76-1.21) | 0.740 | 0.97 (0.35-2.68) | 0.946 |
|  |  | Drinking | 0.70 (0.48-1.03) | 0.069 | 0.72 (0.48-1.08) | 0.112 | 0.00 (0.00-Inf) | 0.976 |
|  |  | Non-drinking | 0.92 (0.75-1.11) | 0.372 | 0.92 (0.75-1.13) | 0.438 | 0.68 (0.26-1.77) | 0.429 |
|  | rs13053714 | Smoking | 1.05 (0.85-1.29) | 0.666 | 1.08 (0.86-1.37) | 0.503 | 0.77 (0.34-1.72) | 0.523 |
|  |  | Non-smoking | 1.12 (0.95-1.33) | 0.178 | 1.17 (0.97-1.40) | 0.100 | 0.81 (0.42-1.59) | 0.549 |
|  |  | Drinking | 1.11 (0.86-1.45) | 0.418 | 1.11 (0.83-1.49) | 0.467 | 1.32 (0.50-3.47) | 0.570 |
|  |  | Non-drinking | 1.08 (0.93-1.26) | 0.313 | 1.14 (0.96-1.35) | 0.124 | 0.63 (0.34-1.16) | 0.138 |
| *PDGFC* | rs1834389 | Smoking | 1.18 (0.98-1.42) | 0.077 | 1.22 (0.99-1.52) | 0.064 | 1.16 (0.65-2.09) | 0.611 |
|  |  | Non-smoking | 1.02 (0.87-1.19) | 0.803 | 1.00 (0.84-1.19) | 0.991 | 1.22 (0.74-2.01) | 0.433 |
|  |  | Drinking | 1.04 (0.82-1.31) | 0.755 | 1.06 (0.81-1.39) | 0.673 | 0.93 (0.44-2.00) | 0.860 |
|  |  | Non-drinking | 1.09 (0.95-1.26) | 0.199 | 1.09 (0.93-1.28) | 0.290 | 1.30 (0.84-2.02) | 0.247 |
|  | rs342309 | Smoking | 1.10 (0.95-1.29) | 0.214 | 1.19 (0.98-1.45) | 0.084 | 0.95 (0.66-1.37) | 0.787 |
|  |  | Non-smoking | 1.08 (0.95-1.22) | 0.251 | 1.12 (0.95-1.31) | 0.181 | 1.03 (0.75-1.41) | 0.850 |
|  |  | Drinking | 0.98 (0.81-1.19) | 0.869 | 1.04 (0.81-1.33) | 0.780 | 0.80 (0.50-1.29) | 0.365 |
|  |  | Non-drinking | 1.12 (1.00-1.25) | 0.059 | 1.17 (1.01-1.35) | 0.033 | 1.07 (0.81-1.41) | 0.653 |
|  | rs6845322 | Smoking | 0.97 (0.85-1.11) | 0.669 | 0.98 (0.79-1.20) | 0.813 | 0.94 (0.74-1.20) | 0.626 |
|  |  | Non-smoking | 0.94 (0.84-1.06) | 0.319 | 0.99 (0.83-1.17) | 0.904 | 0.84 (0.68-1.03) | 0.098 |
|  |  | Drinking | 0.88 (0.74-1.05) | 0.166 | 0.98 (0.75-1.27) | 0.861 | 0.69 (0.50-0.95) | 0.024 |
|  |  | Non-drinking | 0.97 (0.88-1.08) | 0.625 | 0.97 (0.83-1.13) | 0.712 | 0.96 (0.80-1.16) | 0.665 |
| *PDGFD* | rs1053861 | Smoking | 1.00 (0.87-1.15) | 0.999 | 1.07 (0.86-1.32) | 0.555 | 0.92 (0.72-1.17) | 0.496 |
|  |  | Non-smoking | 1.11 (0.99-1.24) | 0.075 | 1.17 (0.98-1.39) | 0.080 | 1.12 (0.92-1.36) | 0.264 |
|  |  | Drinking | 1.05 (0.88-1.25) | 0.597 | 1.25 (0.95-1.64) | 0.115 | 0.86 (0.63-1.19) | 0.369 |
|  |  | Non-drinking | 1.06 (0.96-1.18) | 0.224 | 1.08 (0.92-1.26) | 0.331 | 1.10 (0.92-1.31) | 0.309 |
|  | rs11226185 | Smoking | 0.98 (0.84-1.14) | 0.801 | 1.00 (0.82-1.22) | 0.975 | 0.89 (0.63-1.27) | 0.528 |
|  |  | Non-smoking | 1.09 (0.96-1.23) | 0.186 | 1.08 (0.92-1.27) | 0.346 | 1.21 (0.92-1.60) | 0.179 |
|  |  | Drinking | 1.04 (0.86-1.26) | 0.662 | 1.09 (0.85-1.40) | 0.482 | 0.96 (0.63-1.45) | 0.832 |
|  |  | Non-drinking | 1.05 (0.94-1.17) | 0.436 | 1.04 (0.90-1.20) | 0.583 | 1.11 (0.86-1.43) | 0.422 |
|  | rs4755010 | Smoking | 1.08 (0.93-1.26) | 0.314 | 1.09 (0.90-1.33) | 0.372 | 1.14 (0.80-1.63) | 0.475 |
|  |  | Non-smoking | 0.92 (0.81-1.04) | 0.178 | 0.88 (0.75-1.03) | 0.105 | 0.98 (0.72-1.33) | 0.895 |
|  |  | Drinking | 0.95 (0.79-1.15) | 0.618 | 0.92 (0.72-1.18) | 0.526 | 1.00 (0.64-1.54) | 0.985 |
|  |  | Non-drinking | 0.99 (0.88-1.11) | 0.869 | 0.97 (0.84-1.12) | 0.675 | 1.06 (0.81-1.39) | 0.685 |
| *PDGFRB* | rs6579775 | Smoking | 1.12 (0.94-1.35) | 0.207 | 1.17 (0.95-1.45) | 0.137 | 0.97 (0.54-1.73) | 0.916 |
|  |  | Non-smoking | 0.99 (0.85-1.15) | 0.916 | 1.01 (0.85-1.20) | 0.893 | 0.84 (0.52-1.36) | 0.481 |
|  |  | Drinking | 1.13 (0.89-1.42) | 0.320 | 1.20 (0.92-1.56) | 0.178 | 0.76 (0.34-1.74) | 0.521 |
|  |  | Non-drinking | 1.01 (0.89-1.16) | 0.838 | 1.03 (0.88-1.20) | 0.704 | 0.92 (0.61-1.40) | 0.696 |
|  | rs3828610 | Smoking | 1.12 (0.97-1.29) | 0.109 | 1.14 (0.93-1.41) | 0.204 | 1.20 (0.92-1.55) | 0.174 |
|  |  | Non-smoking | 1.07 (0.95-1.20) | 0.258 | 1.11 (0.94-1.31) | 0.230 | 1.06 (0.86-1.31) | 0.557 |
|  |  | Drinking | **1.26 (1.06-1.50)** | **0.009** | 1.30 (1.00-1.70) | 0.050 | 1.45 (1.06-1.98) | 0.019 |
|  |  | Non-drinking | 1.04 (0.94-1.15) | 0.469 | 1.08 (0.93-1.26) | 0.325 | 1.01 (0.84-1.22) | 0.918 |
|  | rs246390 | Smoking | 0.90 (0.78-1.03) | 0.136 | 0.87 (0.72-1.07) | 0.187 | 0.85 (0.64-1.13) | 0.268 |
|  |  | Non-smoking | 0.89 (0.79-1.00) | 0.045 | 0.88 (0.75-1.03) | 0.107 | 0.81 (0.64-1.03) | 0.089 |
|  |  | Drinking | **0.81 (0.68-0.97)** | **0.024** | 0.76 (0.59-0.97) | 0.030 | 0.77 (0.53-1.10) | 0.149 |
|  |  | Non-drinking | 0.92 (0.83-1.02) | 0.107 | 0.91 (0.79-1.06) | 0.223 | 0.85 (0.69-1.05) | 0.139 |
|  | rs9324641 | Smoking | 1.03 (0.90-1.19) | 0.643 | 0.98 (0.80-1.21) | 0.883 | 1.16 (0.89-1.51) | 0.286 |
|  |  | Non-smoking | 1.01 (0.90-1.13) | 0.889 | 1.02 (0.86-1.21) | 0.828 | 1.00 (0.81-1.24) | 0.986 |
|  |  | Drinking | 1.18 (0.99-1.41) | 0.067 | 1.17 (0.90-1.53) | 0.227 | 1.36 (0.98-1.89) | 0.062 |
|  |  | Non-drinking | 0.98 (0.88-1.09) | 0.694 | 0.97 (0.84-1.13) | 0.700 | 0.98 (0.81-1.18) | 0.811 |

Smoking subgroup analyses were adjusted for age, gender, drinking, hypertension, diabetes, and dyslipidemia.

Drinking subgroup analyses were adjusted for age, gender, smoking, hypertension, diabetes, and dyslipidemia.

Abbreviations: CI, confidence interval; OR, odds ratio; SNP, single nucleotide polymorphism.

**Table S7. Subgroup analyses for the association between pathway SNPs and coronary artery disease by disease statuses**

| **Gene** | **SNP** | **Subgroups** | **Additive Model** | | **Dominant Model** | | **Recessive Model** | |
| --- | --- | --- | --- | --- | --- | --- | --- | --- |
|  |  |  | **OR (95% CI)** | ***P* value** | **OR (95% CI)** | ***P* value** | **OR (95% CI)** | ***P* value** |
| *PDGFA* | rs28472363 | Dyslipidemia | 0.93 (0.69-1.24) | 0.620 | 0.75 (0.50-1.12) | 0.158 | 1.51 (0.75-3.07) | 0.249 |
|  |  | Non-dyslipidemia | 0.99 (0.90-1.09) | 0.884 | 0.99 (0.87-1.13) | 0.864 | 1.00 (0.81-1.23) | 0.969 |
|  |  | Hypertension | 1.05 (0.93-1.18) | 0.432 | 1.03 (0.88-1.21) | 0.717 | 1.15 (0.89-1.49) | 0.272 |
|  |  | Non-hypertension | 0.91 (0.79-1.06) | 0.215 | 0.88 (0.73-1.07) | 0.213 | 0.90 (0.65-1.23) | 0.510 |
|  |  | Diabetes | 1.04 (0.84-1.27) | 0.737 | 0.99 (0.74-1.31) | 0.939 | 1.20 (0.77-1.85) | 0.416 |
|  |  | Non-diabetes | 0.98 (0.88-1.08) | 0.649 | 0.96 (0.84-1.10) | 0.557 | 1.00 (0.80-1.25) | 0.977 |
| *PDGFB* | rs5757573 | Dyslipidemia | 0.84 (0.48-1.47) | 0.545 | 0.89 (0.49-1.60) | 0.691 | 0.23 (0.03-1.98) | 0.180 |
|  |  | Non-dyslipidemia | 0.87 (0.72-1.04) | 0.118 | 0.87 (0.72-1.05) | 0.155 | 0.60 (0.24-1.50) | 0.278 |
|  |  | Hypertension | 0.86 (0.69-1.08) | 0.194 | 0.85 (0.67-1.08) | 0.183 | 0.89 (0.29-2.76) | 0.842 |
|  |  | Non-hypertension | 0.86 (0.66-1.12) | 0.264 | 0.89 (0.67-1.18) | 0.407 | 0.24 (0.04-1.54) | 0.133 |
|  |  | Diabetes | 0.94 (0.66-1.36) | 0.754 | 0.88 (0.59-1.31) | 0.529 | 2.43 (0.45-13.14) | 0.303 |
|  |  | Non-diabetes | 0.84 (0.69-1.02) | 0.074 | 0.86 (0.70-1.06) | 0.155 | 0.21 (0.05-0.85) | 0.028 |
|  | rs13053714 | Dyslipidemia | 0.91 (0.60-1.37) | 0.655 | 0.97 (0.62-1.52) | 0.891 | 0.41 (0.11-1.54) | 0.186 |
|  |  | Non-dyslipidemia | 1.11 (0.97-1.28) | 0.125 | 1.16 (0.99-1.34) | 0.062 | 0.85 (0.49-1.45) | 0.543 |
|  |  | Hypertension | 1.10 (0.92-1.31) | 0.284 | 1.11 (0.92-1.34) | 0.279 | 1.12 (0.56-2.23) | 0.753 |
|  |  | Non-hypertension | 1.10 (0.90-1.34) | 0.364 | 1.19 (0.95-1.49) | 0.128 | 0.49 (0.21-1.14) | 0.100 |
|  |  | Diabetes | 0.96 (0.72-1.29) | 0.802 | 0.98 (0.72-1.34) | 0.892 | 0.74 (0.23-2.34) | 0.606 |
|  |  | Non-diabetes | 1.13 (0.98-1.31) | 0.092 | 1.19 (1.01-1.40) | 0.037 | 0.79 (0.44-1.42) | 0.433 |
| *PDGFC* | rs1834389 | Dyslipidemia | 1.12 (0.76-1.67) | 0.563 | 1.21 (0.77-1.90) | 0.410 | 0.77 (0.25-2.39) | 0.652 |
|  |  | Non-dyslipidemia | 1.08 (0.96-1.23) | 0.201 | 1.08 (0.94-1.25) | 0.296 | 1.26 (0.86-1.87) | 0.239 |
|  |  | Hypertension | 1.16 (0.99-1.35) | 0.065 | 1.15 (0.96-1.37) | 0.127 | 1.51 (0.93-2.46) | 0.098 |
|  |  | Non-hypertension | 0.99 (0.82-1.19) | 0.889 | 1.01 (0.81-1.25) | 0.932 | 0.79 (0.41-1.52) | 0.477 |
|  |  | Diabetes | 1.21 (0.92-1.60) | 0.178 | 1.23 (0.90-1.70) | 0.194 | 1.38 (0.56-3.42) | 0.483 |
|  |  | Non-diabetes | 1.06 (0.93-1.21) | 0.355 | 1.06 (0.91-1.24) | 0.419 | 1.16 (0.76-1.77) | 0.480 |
|  | rs342309 | Dyslipidemia | 0.99 (0.73-1.36) | 0.972 | 1.11 (0.75-1.66) | 0.597 | 0.70 (0.36-1.36) | 0.291 |
|  |  | Non-dyslipidemia | 1.09 (0.99-1.21) | 0.090 | 1.14 (1.00-1.29) | 0.054 | 1.05 (0.82-1.35) | 0.688 |
|  |  | Hypertension | 1.13 (0.99-1.28) | 0.066 | 1.17 (0.99-1.37) | 0.058 | 1.14 (0.84-1.54) | 0.409 |
|  |  | Non-hypertension | 1.02 (0.88-1.20) | 0.768 | 1.09 (0.90-1.32) | 0.393 | 0.83 (0.56-1.22) | 0.341 |
|  |  | Diabetes | 1.14 (0.91-1.43) | 0.267 | 1.17 (0.88-1.55) | 0.273 | 1.17 (0.68-2.04) | 0.568 |
|  |  | Non-diabetes | 1.08 (0.96-1.20) | 0.189 | 1.13 (0.98-1.30) | 0.083 | 0.98 (0.75-1.28) | 0.884 |
|  | rs6845322 | Dyslipidemia | 0.97 (0.73-1.29) | 0.836 | 1.03 (0.67-1.59) | 0.880 | 0.87 (0.53-1.43) | 0.587 |
|  |  | Non-dyslipidemia | 0.95 (0.86-1.04) | 0.256 | 0.97 (0.84-1.11) | 0.637 | 0.88 (0.74-1.04) | 0.140 |
|  |  | Hypertension | 0.98 (0.87-1.10) | 0.696 | 1.05 (0.88-1.25) | 0.580 | 0.87 (0.71-1.07) | 0.178 |
|  |  | Non-hypertension | 0.91 (0.79-1.05) | 0.183 | 0.88 (0.72-1.08) | 0.212 | 0.89 (0.69-1.15) | 0.373 |
|  |  | Diabetes | 0.99 (0.80-1.21) | 0.886 | 1.04 (0.77-1.41) | 0.785 | 0.89 (0.62-1.29) | 0.549 |
|  |  | Non-diabetes | 0.94 (0.86-1.04) | 0.262 | 0.97 (0.83-1.12) | 0.652 | 0.87 (0.73-1.05) | 0.142 |
| *PDGFD* | rs1053861 | Dyslipidemia | 1.08 (0.81-1.44) | 0.592 | 1.24 (0.81-1.88) | 0.316 | 0.94 (0.57-1.55) | 0.822 |
|  |  | Non-dyslipidemia | 1.05 (0.96-1.15) | 0.276 | 1.10 (0.95-1.27) | 0.189 | 1.03 (0.88-1.22) | 0.687 |
|  |  | Hypertension | 1.09 (0.97-1.22) | 0.144 | 1.14 (0.96-1.36) | 0.143 | 1.09 (0.90-1.34) | 0.376 |
|  |  | Non-hypertension | 1.03 (0.90-1.18) | 0.678 | 1.11 (0.90-1.37) | 0.329 | 0.95 (0.75-1.21) | 0.695 |
|  |  | Diabetes | 1.17 (0.96-1.43) | 0.128 | 1.34 (0.99-1.81) | 0.059 | 1.09 (0.77-1.56) | 0.619 |
|  |  | Non-diabetes | 1.04 (0.94-1.15) | 0.423 | 1.08 (0.93-1.26) | 0.307 | 1.02 (0.86-1.21) | 0.816 |
|  | rs11226185 | Dyslipidemia | 0.84 (0.62-1.15) | 0.277 | 0.90 (0.60-1.35) | 0.613 | 0.60 (0.32-1.14) | 0.118 |
|  |  | Non-dyslipidemia | 1.07 (0.97-1.19) | 0.172 | 1.08 (0.94-1.23) | 0.271 | 1.14 (0.91-1.43) | 0.244 |
|  |  | Hypertension | 1.01 (0.89-1.15) | 0.839 | 1.01 (0.86-1.19) | 0.900 | 1.04 (0.78-1.38) | 0.809 |
|  |  | Non-hypertension | 1.09 (0.94-1.26) | 0.272 | 1.11 (0.91-1.35) | 0.295 | 1.12 (0.80-1.56) | 0.517 |
|  |  | Diabetes | 0.96 (0.78-1.19) | 0.731 | 0.88 (0.66-1.17) | 0.373 | 1.21 (0.74-1.97) | 0.450 |
|  |  | Non-diabetes | 1.06 (0.96-1.18) | 0.263 | 1.09 (0.95-1.26) | 0.199 | 1.04 (0.81-1.32) | 0.782 |
|  | rs4755010 | Dyslipidemia | 0.85 (0.62-1.16) | 0.301 | 0.77 (0.52-1.15) | 0.197 | 1.01 (0.46-2.21) | 0.989 |
|  |  | Non-dyslipidemia | 1.00 (0.90-1.11) | 0.975 | 0.98 (0.86-1.12) | 0.774 | 1.06 (0.83-1.35) | 0.641 |
|  |  | Hypertension | 0.99 (0.87-1.13) | 0.908 | 0.96 (0.82-1.13) | 0.606 | 1.12 (0.82-1.51) | 0.481 |
|  |  | Non-hypertension | 0.98 (0.84-1.14) | 0.755 | 0.96 (0.79-1.17) | 0.710 | 0.99 (0.69-1.43) | 0.965 |
|  |  | Diabetes | 0.89 (0.71-1.11) | 0.295 | 0.78 (0.59-1.04) | 0.092 | 1.22 (0.71-2.10) | 0.481 |
|  |  | Non-diabetes | 1.01 (0.91-1.13) | 0.844 | 1.01 (0.88-1.16) | 0.884 | 1.03 (0.79-1.33) | 0.848 |
| *PDGFRB* | rs6579775 | Dyslipidemia | 1.11 (0.75-1.64) | 0.612 | 1.15 (0.75-1.78) | 0.521 | 0.84 (0.23-3.09) | 0.797 |
|  |  | Non-dyslipidemia | 1.03 (0.91-1.16) | 0.617 | 1.06 (0.92-1.22) | 0.424 | 0.89 (0.60-1.31) | 0.541 |
|  |  | Hypertension | 1.00 (0.86-1.16) | 0.978 | 1.01 (0.85-1.20) | 0.919 | 0.95 (0.59-1.53) | 0.847 |
|  |  | Non-hypertension | 1.09 (0.91-1.31) | 0.329 | 1.16 (0.94-1.42) | 0.165 | 0.79 (0.44-1.44) | 0.448 |
|  |  | Diabetes | 1.00 (0.77-1.30) | 0.979 | 0.98 (0.72-1.32) | 0.883 | 1.24 (0.53-2.92) | 0.622 |
|  |  | Non-diabetes | 1.05 (0.92-1.19) | 0.495 | 1.09 (0.94-1.26) | 0.259 | 0.82 (0.54-1.24) | 0.343 |
|  | rs3828610 | Dyslipidemia | **1.47 (1.10-1.97)** | **0.009** | 1.74 (1.15-2.61) | 0.008 | 1.55 (0.89-2.69) | 0.121 |
|  |  | Non-dyslipidemia | 1.05 (0.96-1.16) | 0.269 | 1.07 (0.93-1.23) | 0.320 | 1.07 (0.90-1.27) | 0.429 |
|  |  | Hypertension | 1.05 (0.93-1.17) | 0.439 | 1.05 (0.89-1.24) | 0.581 | 1.08 (0.88-1.33) | 0.462 |
|  |  | Non-hypertension | 1.15 (1.00-1.32) | 0.050 | 1.24 (1.00-1.53) | 0.047 | 1.15 (0.90-1.48) | 0.263 |
|  |  | Diabetes | 1.17 (0.96-1.43) | 0.124 | 1.38 (1.02-1.86) | 0.035 | 1.04 (0.73-1.50) | 0.813 |
|  |  | Non-diabetes | 1.07 (0.97-1.18) | 0.190 | 1.07 (0.92-1.23) | 0.383 | 1.13 (0.94-1.35) | 0.186 |
|  | rs246390 | Dyslipidemia | 0.88 (0.66-1.16) | 0.360 | 0.87 (0.58-1.30) | 0.497 | 0.78 (0.45-1.36) | 0.382 |
|  |  | Non-dyslipidemia | **0.89 (0.81-0.98)** | **0.014** | 0.87 (0.77-1.00) | 0.043 | 0.82 (0.67-1.00) | 0.045 |
|  |  | Hypertension | **0.87 (0.77-0.97)** | **0.016** | 0.82 (0.70-0.97) | 0.020 | 0.84 (0.66-1.06) | 0.131 |
|  |  | Non-hypertension | 0.92 (0.79-1.06) | 0.225 | 0.94 (0.78-1.15) | 0.558 | 0.78 (0.58-1.06) | 0.110 |
|  |  | Diabetes | **0.75 (0.61-0.92)** | **0.005** | 0.70 (0.52-0.92) | 0.013 | 0.65 (0.43-0.99) | 0.044 |
|  |  | Non-diabetes | 0.92 (0.84-1.02) | 0.126 | 0.92 (0.80-1.06) | 0.247 | 0.86 (0.71-1.06) | 0.161 |
|  | rs9324641 | Dyslipidemia | **1.39 (1.03-1.88)** | **0.029** | 1.67 (1.11-2.52) | 0.014 | 1.30 (0.74-2.28) | 0.357 |
|  |  | Non-dyslipidemia | 0.99 (0.90-1.09) | 0.889 | 0.96 (0.84-1.10) | 0.577 | 1.04 (0.87-1.24) | 0.646 |
|  |  | Hypertension | 0.99 (0.88-1.11) | 0.846 | 0.97 (0.82-1.15) | 0.716 | 1.01 (0.81-1.26) | 0.913 |
|  |  | Non-hypertension | 1.06 (0.92-1.22) | 0.394 | 1.06 (0.86-1.30) | 0.598 | 1.13 (0.87-1.46) | 0.366 |
|  |  | Diabetes | 1.14 (0.93-1.40) | 0.202 | 1.42 (1.05-1.90) | 0.021 | 0.90 (0.62-1.30) | 0.581 |
|  |  | Non-diabetes | 0.99 (0.90-1.09) | 0.835 | 0.92 (0.80-1.07) | 0.282 | 1.10 (0.91-1.32) | 0.315 |

Dyslipidemia subgroup analyses were adjusted for age, gender, smoking, drinking, hypertension, and diabetes.

Hypertension subgroup analyses were adjusted for age, gender, smoking, drinking, diabetes, and dyslipidemia.

Diabetes subgroup analyses were adjusted for age, gender, smoking, drinking, hypertension, and dyslipidemia.

Abbreviations: CI, confidence interval; OR, odds ratio; SNP, single nucleotide polymorphism.

**Table S8.** **Weights and risk alleles of SNPs in the PDGF/PDGFRB pathway for coronary artery disease**

| **Gene** | **SNP** | **Allele** | **Weights** | **Risk allele** |
| --- | --- | --- | --- | --- |
| *PDGFA* | rs28472363 | G/A | -0.010548345 | G |
| *PDGFB* | rs5757573 | T/C | -0.151021411 | T |
|  | rs13053714 | G/A | 0.087848192 | A |
| *PDGFC* | rs1834389 | A/C | 0.082474342 | C |
|  | rs342309 | G/A | 0.083029911 | A |
|  | rs6845322 | A/G | -0.05010655 | A |
| *PDGFD* | rs1053861 | C/T | 0.062490436 | T |
|  | rs11226185 | T/C | 0.042180738 | C |
|  | rs4755010 | G/C | -0.014474574 | G |
| *PDGFRB* | rs6579775 | C/T | 0.038263544 | T |
|  | rs3828610 | C/A | 0.083339035 | A |
|  | rs246390 | A/G | -0.12034702 | A |
|  | rs9324641 | C/T | 0.018217539 | T |

Weights were β estimates generated from the multivariable logistic regression analyses of SNPs and coronary artery disease in additive models. Adjusted variables were age, gender, smoking, drinking, hypertension, diabetes, and dyslipidemia.


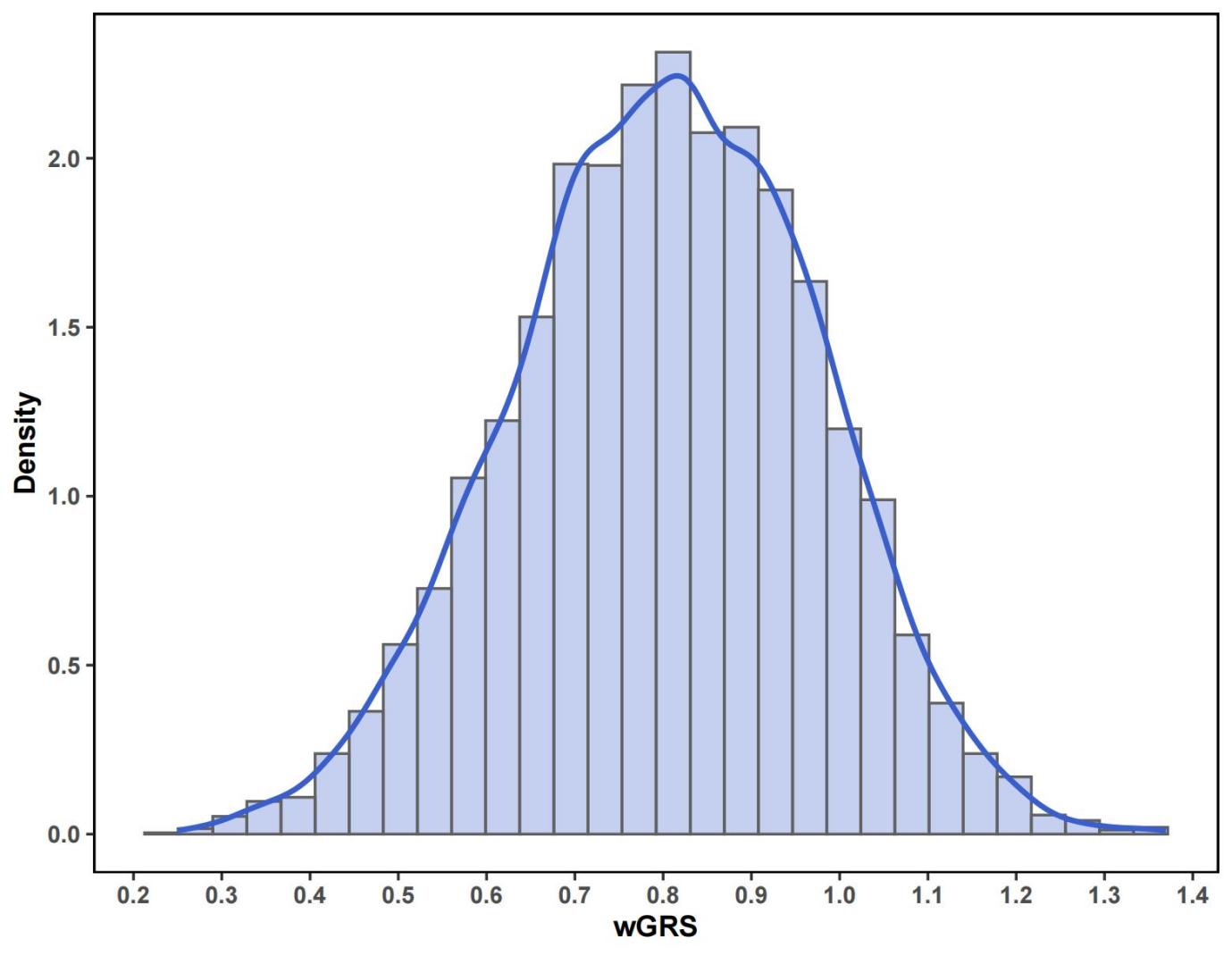
 **Figure S1. Distribution and Kernel density for the weighted genetic risk score (wGRS)**

**Table S9. *PDGF/PDGFRB* mRNA expression comparison between acute myocardial infarction cases and controls**

| **Gene** | **Cases (n = 306)** | **Controls (n = 306)** | **Fold Change** | **Z** | ***P*** |
| --- | --- | --- | --- | --- | --- |
| *PDGFA*, Median (Q1, Q3) | 0.733 (0.401, 1.494) | 1.038 (0.612, 1.632) | 0.706 | 3.771 | < 0.001 |
| *PDGFB*, Median (Q1, Q3) | 0.566 (0.235, 1.273) | 0.968 (0.566, 1.712) | 0.585 | 5.649 | < 0.001 |
| *PDGFC*, Median (Q1, Q3) | 0.666 (0.340, 1.109) | 0.984 (0.656, 1.538) | 0.677 | 6.352 | < 0.001 |
| *PDGFD*, Median (Q1, Q3) | 0.411 (0.182, 0.738) | 1.024 (0.577, 1.574) | 0.401 | 11.611 | < 0.001 |
| *PDGFRB*, Median (Q1, Q3) | 0.349 (0.142, 0.672) | 1.006 (0.612, 1.658) | 0.347 | 12.642 | < 0.001 |

Data are medians and interquartile range.

Mann-Whitney U test was used for comparison.

Abbreviations: Q1, the first (25%) quartile; Q3, the third (75%) quartile.

**Table S10.** ***PDGF/PDGFRB* mRNA expression comparison between acute myocardial infarction cases at admission and at discharge**

| **Gene mRNA** | **Admitted AMI cases**  **(n = 250)** | **Discharged AMI cases**  **(n = 250)** | **Fold Change** | **Z** | ***P*** |
| --- | --- | --- | --- | --- | --- |
| *PDGFA*, Median (Q1, Q3) | 0.733 (0.401, 1.513) | 0.945 (0.566, 1.571) | 1.289 | 1.473 | 0.141 |
| *PDGFB*, Median (Q1, Q3) | 0.545 (0.235, 1.261) | 0.965 (0.540, 1.535) | 1.771 | 3.035 | 0.002 |
| *PDGFC*, Median (Q1, Q3) | 0.634 (0.324, 1.138) | 0.909 (0.561, 1.515) | 1.434 | 5.048 | < 0.001 |
| *PDGFD*, Median (Q1, Q3) | 0.458 (0.178, 0.803) | 0.996 (0.599, 1.644) | 2.175 | 8.820 | < 0.001 |
| *PDGFRB*, Median (Q1, Q3) | 0.342 (0.135, 0.661) | 1.019 (0.573, 1.876) | 2.980 | 10.321 | < 0.001 |

Data are medians and interquartile range.

Wilcoxon signed rank test was used for paired comparison.

Abbreviations: AMI, acute myocardial infarction; Q1, the first (25%) quartile; Q3, the third (75%) quartile.

**Table S11. Association of *PDGFs* and *PDGFRB* mRNA expression with acute myocardial infarction**

| **Gene** | **Quartile** | **Unadjusted Model** | | **Adjusted Model** | |
| --- | --- | --- | --- | --- | --- |
|  |  | **OR (95% CI)** | ***P* value** | **OR (95% CI)** | ***P* value** |
| *PDGFA* | Q4 | Reference | - | Reference | - |
|  | Q1 | 1.892 (1.202-2.994) | 0.006 | 1.905 (1.149-3.177) | 0.013 |
|  | Q2 | 1.369 (0.874-2.151) | 0.170 | 1.370 (0.828-2.273) | 0.221 |
|  | Q3 | 0.686 (0.433-1.081) | 0.105 | 0.632 (0.376-1.056) | 0.081 |
| *PDGFB* | Q4 | Reference | - | Reference | - |
|  | Q1 | 3.484 (2.172-5.658) | <0.001 | 3.625 (2.139-6.233) | <0.001 |
|  | Q2 | 1.301 (0.830-2.044) | 0.252 | 1.394 (0.837-2.329) | 0.203 |
|  | Q3 | 0.699 (0.439-1.107) | 0.128 | 0.705 (0.416-1.188) | 0.190 |
| *PDGFC* | Q4 | Reference | - | Reference | - |
|  | Q1 | 4.058 (2.529-6.597) | <0.001 | 3.949 (2.329-6.793) | <0.001 |
|  | Q2 | 1.749 (1.111-2.765) | 0.016 | 1.687 (1.021-2.805) | 0.042 |
|  | Q3 | 1.057 (0.667-1.676) | 0.814 | 1.097 (0.656-1.835) | 0.725 |
| *PDGFD* | Q4 | Reference | - | Reference | - |
|  | Q1 | 18.366 (10.524-33.169) | <0.001 | 18.085 (9.813-34.598) | <0.001 |
|  | Q2 | 5.328 (3.236-8.957) | <0.001 | 5.060 (2.924-8.943) | <0.001 |
|  | Q3 | 2.609 (1.577-4.385) | <0.001 | 2.209 (1.273-3.886) | 0.005 |
| *PDGFRB* | Q4 | Reference | - | Reference | - |
|  | Q1 | 22.037 (12.427-40.615) | <0.001 | 27.784 (14.393-56.229) | <0.001 |
|  | Q2 | 6.532 (3.943-11.069) | <0.001 | 8.185 (4.610-14.959) | <0.001 |
|  | Q3 | 2.173 (1.300-3.686) | 0.003 | 2.333 (1.309-4.229) | 0.005 |

Adjusted model: adjusted for age, gender, smoking, drinking, hypertension, diabetes, and dyslipidemia.

Abbreviations: CI, confidence interval; OR, odds ratio; Q1, the first quartile, P_0_-P_25_; Q2, the second quartile, P_25_-P_50_; Q3, the third quartile, P_50_-P_75_; Q4, the fourth quartile, P_75_-P_100_.

**Table S12. *PDGFs* and *PDGFRB* mRNA expression levels by SNP genotypes**

| **Gene** | **SNP** | **Controls** | |  | **Cases** | |
| --- | --- | --- | --- | --- | --- | --- |
|  |  | **Genotype** | ***PDGF* mRNA, Median (Q1, Q3)** |  | **Genotype** | ***PDGF* mRNA, Median (Q1, Q3)** |
| *PDGFA* | rs28472363 | GG (n = 117) | 1.040 (0.593, 1.683) |  | GG (n = 141) | 0.796 (0.472, 1.512) |
|  |  | GA (n = 131) | 1.084 (0.654, 1.576) |  | GA (n = 112) | 0.714 (0.380, 1.544) |
|  |  | AA (n = 36) | 1.004 (0.741, 1.515) |  | AA (n = 27) | 0.620 (0.322, 0.879) |
|  |  | *P* | 0.711 |  | *P* | 0.237 |
|  |  | *P_trend_* | 0.640 |  | *P_trend_* | 0.181 |
| *PDGFB* | rs5757573 | TT (n = 246) | 0.956 (0.558, 1.672) |  | TT (n = 242) | 0.593 (0.241, 1.261) |
|  |  | TC (n = 37) | 1.056 (0.628, 1.446) |  | TC (n = 35) | 0.388 (0.208, 0.874) |
|  |  | CC (n = 1) | 5.101 (5.101, 5.101) |  | CC (n = 3) | 0.177 (0.111, 0.267) |
|  |  | *P* | 0.302 |  | *P* | 0.066 |
|  |  | *P_trend_* | 0.700 |  | *P_trend_* | 0.052 |
|  | rs13053714 | GG (n = 206) | 0.939 (0.571, 1.495) |  | GG (n = 215) | 0.542 (0.233, 1.112) |
|  |  | GA (n = 73) | 0.982 (0.601, 2.074) |  | GA (n = 63) | 0.610 (0.231, 1.339) |
|  |  | AA (n = 5) | 0.582 (0.555, 1.439) |  | AA (n = 2) | 0.847 (0.717, 0.977) |
|  |  | *P* | 0.377 |  | *P* | 0.691 |
|  |  | *P_trend_* | 0.347 |  | *P_trend_* | 0.477 |
| *PDGFC* | rs1834389 | AA (n = 203) | 0.988 (0.621, 1.510) |  | AA (n = 196) | 0.726 (0.391, 1.186) |
|  |  | AC (n = 74) | 0.984 (0.690, 1.417) |  | AC (n = 72) | 0.612 (0.265, 1.050) |
|  |  | CC (n = 7) | 1.374 (0.772, 2.896) |  | CC (n = 12) | 0.548 (0.426, 0.806) |
|  |  | *P* | 0.450 |  | *P* | 0.282 |
|  |  | *P_trend_* | 0.795 |  | *P_trend_* | 0.117 |
|  | rs342309 | GG (n = 157) | 0.954 (0.580, 1.495) |  | GG (n = 139) | 0.702 (0.395, 1.116) |
|  |  | GA (n = 109) | 1.005 (0.688, 1.538) |  | GA (n = 122) | 0.646 (0.299, 1.173) |
|  |  | AA (n = 18) | 0.959 (0.669, 1.538) |  | AA (n = 19) | 0.755 (0.371, 0.889) |
|  |  | *P* | 0.777 |  | *P* | 0.623 |
|  |  | *P_trend_* | 0.530 |  | *P_trend_* | 0.347 |
|  | **rs6845322** | AA (n = 96) | 1.196 (0.736, 1.745) |  | AA (n = 86) | 0.723 (0.349, 1.133) |
|  |  | AG (n = 137) | 0.938 (0.628, 1.429) |  | AG (n = 138) | 0.646 (0.370, 1.107) |
|  |  | GG (n = 51) | 0.774 (0.540, 1.124) |  | GG (n = 56) | 0.665 (0.412, 1.112) |
|  |  | *P* | **0.002** |  | *P* | 0.892 |
|  |  | *P_trend_* | **3.4****×10^-4^** |  | *P_trend_* | 0.654 |
| *PDGFD* | rs1053861 | CC (n = 92) | 1.008 (0.542, 1.459) |  | CC (n = 73) | 0.340 (0.178, 0.671) |
|  |  | CT (n = 139) | 1.024 (0.634, 1.618) |  | CT (n = 149) | 0.431 (0.181, 0.823) |
|  |  | TT (n = 53) | 1.042 (0.463, 1.579) |  | TT (n = 58) | 0.430 (0.194, 0.672) |
|  |  | *P* | 0.747 |  | *P* | 0.347 |
|  |  | *P_trend_* | 0.555 |  | *P_trend_* | 0.515 |
|  | rs11226185 | TT (n = 150) | 1.015 (0.635, 1.411) |  | TT (n = 136) | 0.404 (0.177, 0.656) |
|  |  | TC (n = 112) | 1.108 (0.575, 1.841) |  | TC (n = 117) | 0.369 (0.198, 0.698) |
|  |  | CC (n = 22) | 0.742 (0.435, 1.324) |  | CC (n = 27) | 0.758 (0.204, 0.948) |
|  |  | *P* | 0.156 |  | *P* | 0.341 |
|  |  | *P_trend_* | 0.956 |  | *P_trend_* | 0.234 |
|  | rs4755010 | GG (n = 140) | 1.033 (0.602, 1.698) |  | GG (n = 133) | 0.510 (0.202, 0.831) |
|  |  | GC (n = 118) | 1.011 (0.516, 1.439) |  | GC (n = 120) | 0.332 (0.173, 0.601) |
|  |  | CC (n = 26) | 1.088 (0.654, 2.273) |  | CC (n = 27) | 0.498 (0.182, 0.682) |
|  |  | *P* | 0.220 |  | *P* | 0.071 |
|  |  | *P_trend_* | 0.738 |  | *P_trend_* | 0.067 |
| *PDGFRB* | rs6579775 | CC (n = 195) | 0.991 (0.614, 1.648) |  | CC (n = 86) | 0.302 (0.143, 0.621) |
|  |  | CT (n = 80) | 1.077 (0.636, 1.622) |  | CA (n = 142) | 0.329 (0.142, 0.639) |
|  |  | TT (n = 9) | 1.017 (0.828, 1.716) |  | AA (n = 52) | 0.418 (0.125, 0.872) |
|  |  | *P* | 0.948 |  | *P* | 0.698 |
|  |  | *P_trend_* | 0.791 |  | *P_trend_* | 0.905 |
|  | rs3828610 | CC (n = 99) | 1.057 (0.607, 1.744) |  | CC (n = 86) | 0.302 (0.143, 0.621) |
|  |  | CA (n = 134) | 1.010 (0.609, 1.708) |  | CA (n = 142) | 0.329 (0.142, 0.639) |
|  |  | AA (n = 51) | 1.017 (0.686, 1.344) |  | AA (n = 52) | 0.418 (0.125, 0.872) |
|  |  | *P* | 0.944 |  | *P* | 0.732 |
|  |  | *P_trend_* | 0.803 |  | *P_trend_* | 0.675 |
|  | rs246390 | AA (n = 120) | 1.021 (0.654, 1.713) |  | AA (n = 181) | 0.353 (0.142, 0.742) |
|  |  | AG (n = 130) | 1.003 (0.623, 1.444) |  | AG (n = 74) | 0.282 (0.13, 0.529) |
|  |  | GG (n = 34) | 1.043 (0.544, 1.735) |  | GG (n = 25) | 0.325 (0.161, 0.742) |
|  |  | *P* | 0.711 |  | *P* | 0.470 |
|  |  | *P_trend_* | 0.454 |  | *P_trend_* | 0.492 |
|  | rs9324641 | CC (n = 97) | 1.057 (0.498, 1.748) |  | CC (n = 95) | 0.315 (0.159, 0.691) |
|  |  | CT (n = 136) | 1.006 (0.615, 1.716) |  | CT (n = 134) | 0.319 (0.136, 0.594) |
|  |  | TT (n = 51) | 1.038 (0.675, 1.344) |  | TT (n = 51) | 0.432 (0.125, 0.853) |
|  |  | *P* | 0.892 |  | *P* | 0.476 |
|  |  | *P_trend_* | 0.931 |  | *P_trend_* | 0.738 |

Kruskal-Wallis test was used for the comparison among genotypes.

Jonckheere-Terpstra test was used for the trend analysis.

Abbreviations: CAD, coronary artery disease; Q1, the first (25%) quartile; Q3, the third (75%) quartile; SNP, single nucleotide polymorphism.

**Table S13. Expression quantitative trait loci (eQTL) analysis of 12 SNPs and *PDGFs/PDGFRB* expressions**

| **SNP** | **Database** | **Gene ID** | **Symbol** | **Tested Allele** | ***P*** | **Stats** | **FDR** | **chr** | **pos** |
| --- | --- | --- | --- | --- | --- | --- | --- | --- | --- |
| rs28472363 | GTEx/v8 | ENSG00000197461 | PDGFA | G | 3.07E-05 | 0.155028 | 7.02E-06 | 7 | 551122 |
| rs5757573 | GTEx/v8 | ENSG00000100311 | PDGFB | T | 1.04E-13 | 0.281271 | 9.55E-11 | 22 | 39633622 |
| rs1834389 | eQTLGen | ENSG00000145431 | PDGFC | C | 3.04E-42 | -13.6202 | 0.00E+00 | 4 | 157718612 |
| rs342309 | eQTLGen | ENSG00000145431 | PDGFC | A | 4.49E-40 | -13.2503 | 0.00E+00 | 4 | 157811441 |
| rs6845322 | eQTLGen | ENSG00000145431 | PDGFC | G | 2.14E-06 | -4.7396 | 6.24E-03 | 4 | 157884105 |
| rs1053861 | eQTLGen | ENSG00000170962 | PDGFD | T | 5.57E-42 | -13.5756 | 0.00E+00 | 11 | 103778154 |
| rs11226185 | eQTLGen | ENSG00000170962 | PDGFD | C | 1.20E-19 | 9.0691 | 0.00E+00 | 11 | 103991641 |
| rs4755010 | PsychENCODE | ENSG00000170962 | PDGFD | G | 1.32E-24 | 0.157228 | 9.80E-22 | 11 | 104034148 |
| rs246390 | GTEx/v8 | ENSG00000113721 | PDGFRB | G | 6.85E-08 | 0.154608 | 1.89E-10 | 5 | 149496321 |
| rs9324641 | GTEx/v8 | ENSG00000113721 | PDGFRB | C | 1.07E-07 | -0.503139 | 2.08E-04 | 5 | 149527844 |
| rs6579775 | GTEx/v8 | ENSG00000113721 | PDGFRB | T | 8.71E-07 | -0.531536 | 2.08E-04 | 5 | 149533848 |
| rs3828610 | GTEx/v8 | ENSG00000113721 | PDGFRB | C | 1.16E-07 | -0.495808 | 2.08E-04 | 5 | 149535625 |

Based on the common public eQTL database, 12 SNPs in this study except rs13053714 are recorded to be cis-eQTL, which have significant associations with their corresponding nearby genes.

GTEx: Genotype-Tissue Expression Program; eQTLGen: eQTLGen Consortium.

FDR, false discovery rate; chr, chromosome; pos, position.

**
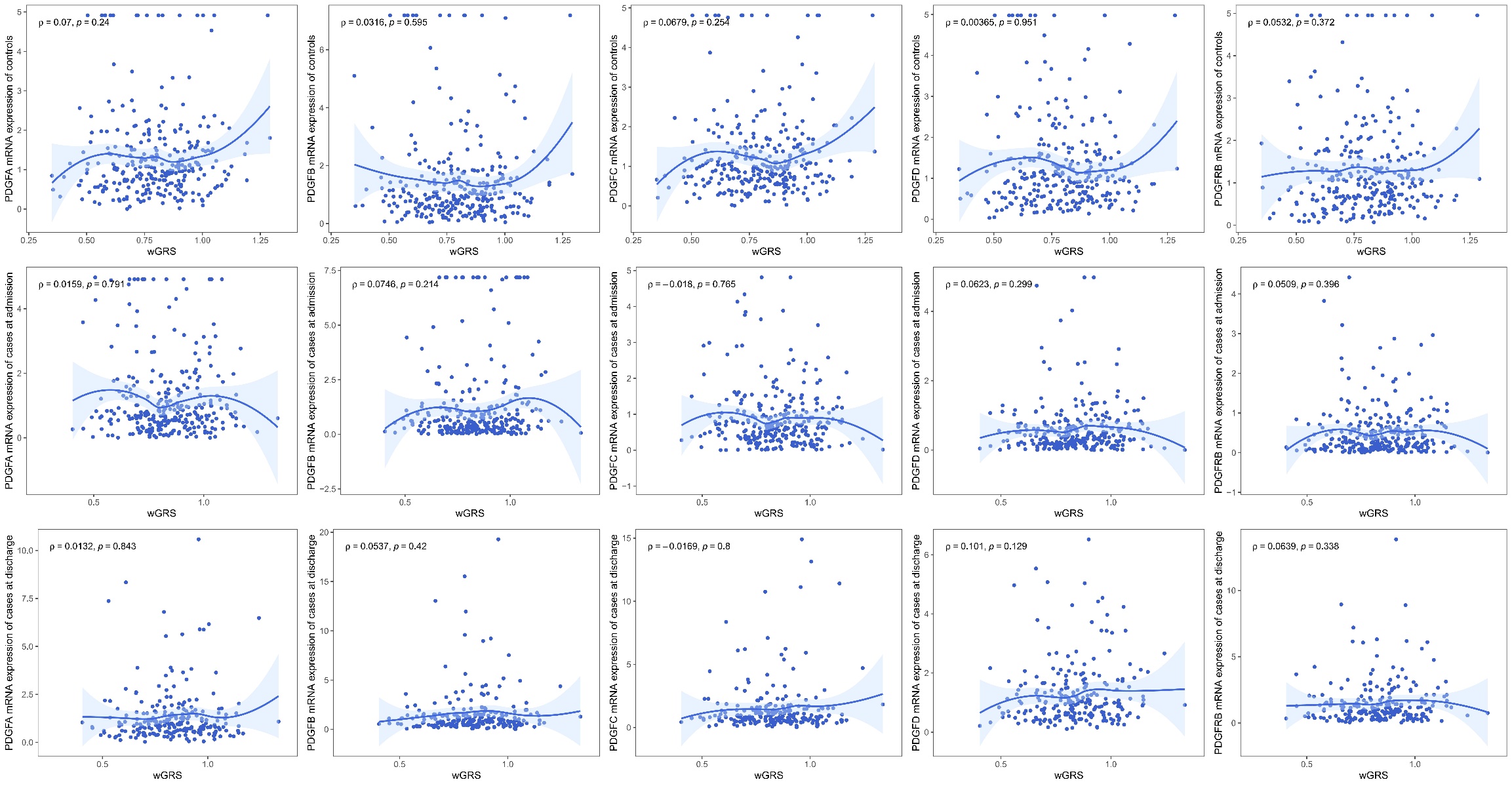
Figure S2.** **Spearman correlation** **between wGRS and *PDGF/PDGFRB* mRNA expression levels**

Association curves are fitted by the locally weighted scatterplot smoothing method.

Abbreviations: wGRS, weighted genetic risk score.

**Table S14.** **Discrimination performance of wGRS, *PDGF/PDGFRB* mRNAs, and traditional risk factors for coronary artery disease**

| **Model** | **N** | **AUC (95% CI)** | **Optimal Cutoff** | **Specificity** | **Sensitivity** | ***P**** |
| --- | --- | --- | --- | --- | --- | --- |
| TRF | 6409 | 0.837 (0.827-0.847) | 0.460 | 0.850 | 0.674 | reference |
| wGRS | 6409 | 0.543 (0.529-0.557) | 0.487 | 0.502 | 0.568 | <0.001 |
| *PDGFA* mRNA | 564 | 0.586 (0.539-0.633) | 0.836 | 0.644 | 0.554 | <0.001^a^ |
| *PDGFB* mRNA | 564 | 0.644 (0.597-0.690) | 0.553 | 0.775 | 0.504 | <0.001^b^ |
| *PDGFC* mRNA | 564 | 0.644 (0.598-0.689) | 0.653 | 0.761 | 0.482 | <0.001^c^ |
| *PDGFD* mRNA | 564 | 0.771 (0.732-0.809) | 0.783 | 0.648 | 0.779 | <0.001^d^ |
| *PDGFRB* mRNA | 564 | 0.797 (0.760-0.834) | 0.564 | 0.796 | 0.693 | <0.001^e^ |
| wGRS+5 gene mRNA | 564 | 0.816 (0.781-0.851) | 0.555 | 0.761 | 0.761 | 0.034 |
| TRF+wGRS | 6409 | 0.838 (0.829-0.848) | 0.524 | 0.898 | 0.626 | 0.117 |
| TRF+wGRS+*PDGFA* mRNA | 564 | 0.868 (0.838-0.897) | 0.421 | 0.806 | 0.779 | 0.617 |
| TRF+wGRS+*PDGFB* mRNA | 564 | 0.865 (0.836-0.895) | 0.416 | 0.796 | 0.771 | 0.862 |
| TRF+wGRS+*PDGFC* mRNA | 564 | 0.880 (0.852-0.908) | 0.507 | 0.891 | 0.732 | 0.032 |
| TRF+wGRS+*PDGFD* mRNA | 564 | 0.890 (0.863-0.917) | 0.470 | 0.856 | 0.782 | 0.001 |
| TRF+wGRS+*PDGFRB* mRNA | 564 | 0.919 (0.896-0.942) | 0.431 | 0.835 | 0.864 | <0.001 |
| TRF+wGRS+5 gene mRNA | 564 | 0.921 (0.898-0.943) | 0.422 | 0.827 | 0.875 | <0.001 |

*: DeLong test was used for AUC comparison with uniform sample size.

a: Compared to model TRF+wGRS+PDGFA mRNA.

b: Compared to model TRF+wGRS+PDGFB mRNA.

c: Compared to model TRF+wGRS+PDGFC mRNA.

d: Compared to model TRF+wGRS+PDGFD mRNA.

e: Compared to model TRF+wGRS+PDGFRB mRNA.

Traditional risk factors included age, gender, smoking, drinking, hypertension, diabetes, and dyslipidemia.

Abbreviations: AUC, area under the curve; CI, confidence interval; TRF, traditional risk factors; wGRS, weighted genetic risk score.
